# Viral sepsis: diagnosis, clinical features, pathogenesis, and clinical considerations

**DOI:** 10.1186/s40779-024-00581-0

**Published:** 2024-12-16

**Authors:** Ji-Qian Xu, Wan-Ying Zhang, Jia-Ji Fu, Xiang-Zhi Fang, Cheng-Gang Gao, Chang Li, Lu Yao, Qi-Lan Li, Xiao-Bo Yang, Le-Hao Ren, Hua-Qing Shu, Ke Peng, Ying Wu, Ding-Yu Zhang, Yang Qiu, Xi Zhou, Yong-Ming Yao, You Shang

**Affiliations:** 1 Department of Critical Care Medicine, Union Hospital, Tongji Medical College, Huazhong University of Science and Technology, Wuhan, 430022 China; 2 State Key Laboratory of Virology, Center for Antiviral Research, Wuhan Institute of Virology, Chinese Academy of Sciences, Wuhan, 43007 China; 3 State Key Laboratory of Virology and Hubei Province Key Laboratory of Allergy and Immunology, Institute of Medical Virology, TaiKang Medical School, Wuhan University, Wuhan, 430072 China; 4 Translational Medicine Research Center, Medical Innovation Research Division and the Fourth Medical Center of Chinese, PLA General Hospital, Beijing, 100853 China

**Keywords:** Viral sepsis, Epidemiology, Definition, Immunopathology, Treatment strategies

## Abstract

Sepsis, characterized as life-threatening organ dysfunction resulting from dysregulated host responses to infection, remains a significant challenge in clinical practice. Despite advancements in understanding host-bacterial interactions, molecular responses, and therapeutic approaches, the mortality rate associated with sepsis has consistently ranged between 10 and 16%. This elevated mortality highlights critical gaps in our comprehension of sepsis etiology. Traditionally linked to bacterial and fungal pathogens, recent outbreaks of acute viral infections, including Middle East respiratory syndrome coronavirus (MERS-CoV), influenza virus, and severe acute respiratory syndrome coronavirus 2 (SARS-CoV-2), among other regional epidemics, have underscored the role of viral pathogenesis in sepsis, particularly when critically ill patients exhibit classic symptoms indicative of sepsis. However, many cases of viral-induced sepsis are frequently underdiagnosed because standard evaluations typically exclude viral panels. Moreover, these viruses not only activate conventional pattern recognition receptors (PRRs) and retinoic acid-inducible gene-I (RIG-I)-like receptors (RLRs) but also initiate primary antiviral pathways such as cyclic guanosine monophosphate adenosine monophosphate (GMP-AMP) synthase (cGAS)-stimulator of interferon genes (STING) signaling and interferon response mechanisms. Such activations lead to cellular stress, metabolic disturbances, and extensive cell damage that exacerbate tissue injury while leading to a spectrum of clinical manifestations. This complexity poses substantial challenges for the clinical management of affected cases. In this review, we elucidate the definition and diagnosis criteria for viral sepsis while synthesizing current knowledge regarding its etiology, epidemiology, and pathophysiology, molecular mechanisms involved therein as well as their impact on immune-mediated organ damage. Additionally, we discuss clinical considerations related to both existing therapies and advanced treatment interventions, aiming to enhance the comprehensive understanding surrounding viral sepsis.

## Background

Sepsis, as defined by Sepsis-3.0, is characterized by life-threatening organ dysfunction resulting from a dysregulated host response to infection [1, 2]. Clinically, it manifests as an acute elevation in the Sequential Organ Failure Assessment (SOFA) score of ≥ 2, and timely interventions can potentially mitigate organ dysfunctions through effective infection control, hemodynamic stabilization, and supportive care for affected organs [1, 3]. Despite these therapeutic measures, the mortality rate associated with sepsis remains approximately 10 to 16% [1, 4], underscoring the urgent need for a deeper understanding of its etiological factors.

Bacterial, fungal, protozoa, and viral pathogens can all induce sepsis [5], which remains a leading cause of mortality among patients infected with these agents, including those suffering from visceral leishmaniasis. However, detection rates for these pathogens in septic cases range from 25 to 40%, with bacterial detection being the most prevalent [6]. Bacteria are typically identified via culture and advanced molecular techniques, while fungi are detected through serological assays and culture methods. Conversely, viral sepsis is often underdiagnosed due to standard evaluations not routinely incorporating viral panels. This diagnostic gap became particularly evident during pandemics of acute viral infections such as coronavirus disease 2019 (COVID-19), Middle East respiratory syndrome (MERS), and influenza. Many critically ill patients exhibited classic symptoms of sepsis similar to those seen in bacterial infections despite the absence of bacterial or fungal co-infections [7–9]. Notably, estimates indicate that mortality associated with viral sepsis is significantly higher, such as COVID-19-associated sepsis mortality ranging from 22 to 40% [10, 11], highlighting the critical role of viruses in the etiology of sepsis [7].

Viruses exhibit distinct mechanisms for eliciting immune responses compared to bacteria and fungi. While bacteria and fungi utilize endotoxins, superantigens, and various toxins, viruses primarily exploit their nucleic acid genomes or protein structures [5]. Viruses may infect non-immune host cells, resulting in cellular damage that amplifies both immune responses and tissue injury. Moreover, viruses often target immune cells (e.g., T and B cells) to stimulate the humoral immune response [4], leading to increased antibody production that may further exacerbate tissue damage. Additionally, viruses can modulate natural killer (NK) cells, crucial components of the immune defense, which may inadvertently harm uninfected tissues. Upon invading host cells, viruses engage traditional pattern recognition receptor (PRR) or retinoic acid-inducible gene-I (RIG-I)-like receptors (RLRs) signaling pathways. They also enhance first-line antiviral defenses such as cyclic guanosine monophosphate adenosine monophosphate (GMP-AMP) synthase (cGAS)-stimulator of interferon genes (STING) and interferon signaling that lead to cellular stress, metabolic disruptions, and cell death, resulting in diverse clinical manifestations [4]. These intricate pathophysiological processes contribute additional layers of complexity to virus-induced sepsis.

Here, we characterize sepsis triggered by several common acute viral infections, encompassing the definition, diagnosis, epidemiology, organ damage, and immune pathogenesis. We also discuss the clinical considerations regarding therapeutic interventions for these conditions. This comprehensive analysis aims to enhance our understanding of virus-induced sepsis and provide insights into relevant therapeutic strategies.

## Definition and diagnosis of viral sepsis

### Definition

**Fig. 1:**
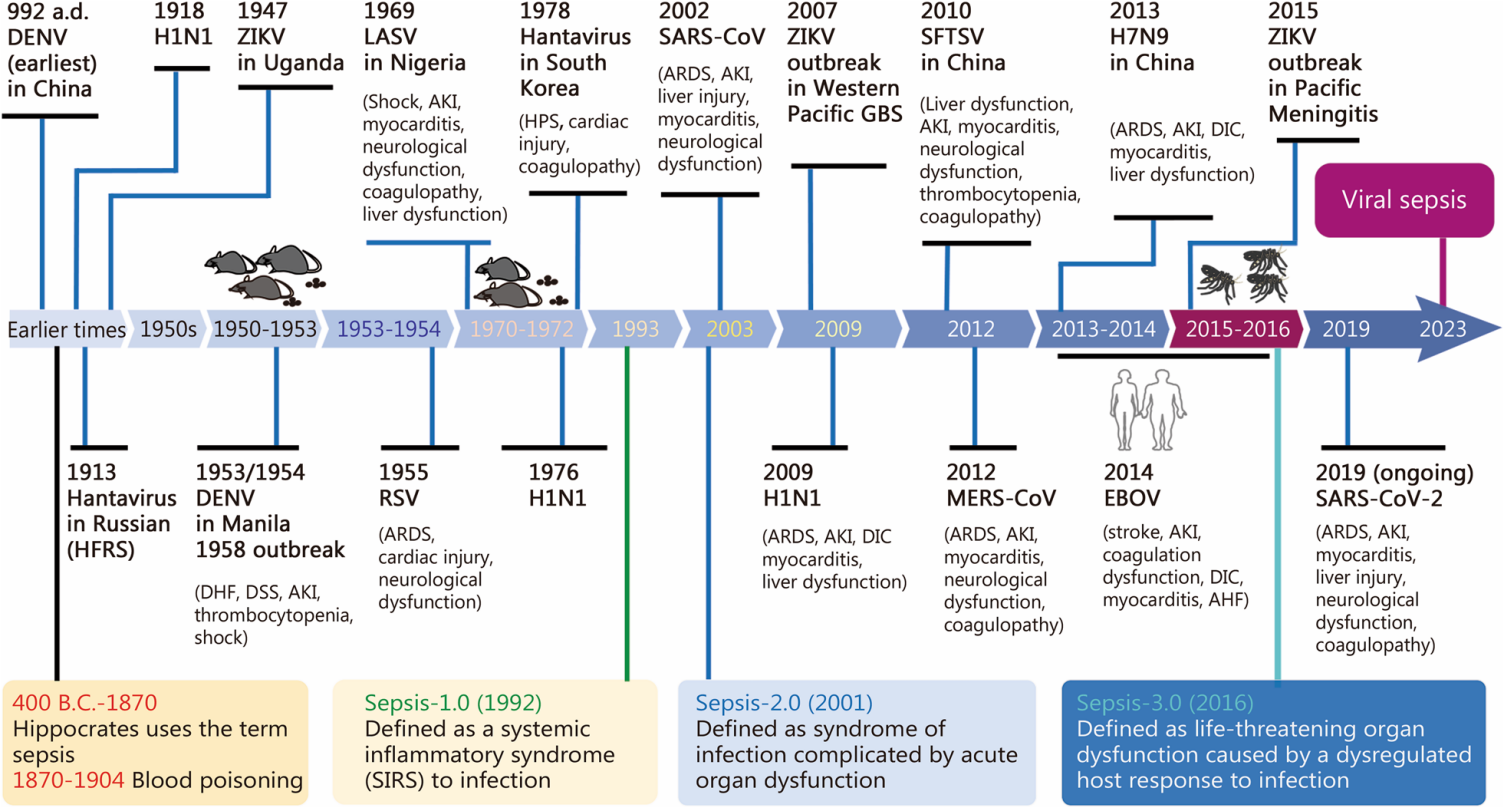
Timeline of virus outbreak associated with organ dysfunction and the evolution of sepsis definition. Reports of illnesses clinically compatible with dengue fever can be traced back to a Chinese medical encyclopedia from 992 a.d. The first documented epidemic of dengue virus (DENV) occurred between 1953 and 1954, presenting clinical symptoms involving DHF, DSS, and AKI. RSV was first identified in infants in 1955, with critical cases progressing to ARDS. Hantavirus infection causing HFRS was first recorded in Russian clinical records as early as 1913, while HPS was identified for the first time in 1993. Lassa virus (LASV) was recognized as the causative agent of Lassa fever in 1969. In 2003, SARS-CoV triggered an epidemic outbreak that resulted in ARDS and myocarditis. Influenza viruses have caused several global outbreaks, including H1N1 pandemics in 1918, 1976, and 2009, along with H7N9 outbreaks in 2013. All of these strains can capable of inducing ARDS, myocarditis, AKI, and DIC. Dabie bandavirus was discovered for the first time in 2010 and is linked to SFTS and DIC. MERS-CoV, which can induce ARDS and myocarditis, emerged in 2012. Zika virus (ZIKV) has been associated with GBS since its identification in 2007 and meningitis during the period from 2015 to 2016. Between 2013 and 2016, Ebola virus (EBOV) led to a significant outbreak characterized by clinical manifestations including DIC, myocarditis, and acute hepatic failure. Most recently, SARS-CoV-2 has instigated a global pandemic since its emergence in 2019, leading to multiple organ dysfunctions such as ARDS, myocarditis, acute hepatic failure, AKI, and DIC. a.d. Anno Domini, DHF dengue hemorrhagic fever, DSS dengue shock syndrome, AKI acute kidney injury, RSV respiratory syncytial virus, HFRS haemorrhagic fever with renal syndrome, ARDS acute respiratory distress syndrome, DIC disseminated intravascular coagulation, MERS-CoV Middle East respiratory syndrome coronavirus, SFTS severe fever with thrombocytopenia syndrome, HPS hantavirus pulmonary syndrome, SARS-CoV-2 severe acute respiratory syndrome coronavirus 2, AHF acute heart failure

Sepsis-3.0, the most recent international consensus on sepsis, represents a substantial advancement in its definition by emphasizing organ dysfunction and dysregulated immune responses [1, 2]. However, it does not fully encompass the diversity caused by specific pathogens or host factors (Fig. 1). This distinction is crucial, as different pathogens may trigger similar immune responses and cause organ damage but necessitate distinct therapeutic approaches. Consequently, terms such as bacterial and fungal sepsis have been introduced to highlight the importance of pathogen-specific identification and tailored treatment strategies [4].

Defining viral sepsis poses significant challenges due to the intricate interactions between viruses and host cells. Chronic viral infections, such as hepatitis B virus (HBV) and hepatitis C virus (HCV) progress slowly, resulting in minimal damage during the initial stages. In contrast, latent infections like herpes simplex virus (HSV), cytomegalovirus (CMV), Epstein-Barr virus (EBV), and human immunodeficiency virus (HIV) can remain asymptomatic for extended periods. Ultimately, these infections may present with high SOFA scores that fulfill the Sepsis-3.0 criteria in their terminal stages, typically indicating end-stage disease rather than sepsis caused by irreversible organ damage.

In HIV patients, elevated cytokine levels of interleukin (IL)-1, IL-6, IL-10, and tumor necrosis factor (TNF) are associated with the pathogenesis of sepsis in acute cases. These elevated cytokine levels arise from a primed immune state that leads to hyper-responsiveness to translocated microbial products from the gut [12]. Additionally, HIV can severely impair the immune system, increasing vulnerability to invasive infections; however, this immune system is only partially restored by combination antiretroviral therapy [12]. Consequently, when patients with HIV/acquired immunodeficiency syndrome meet the Sepsis-3.0 criteria, their condition should not be solely attributed to viral sepsis.

Moreover, patients with chronic or latent viral infections, in the absence of bacterial, fungal, or parasitic infections, may experience acute deterioration due to impaired immune responses, leading to distinct organ dysfunction. In such cases, rapid changes in SOFA scores and timely interventions can effectively reverse this dysfunction, similar to bacterial sepsis. These situations should be recognized as sepsis induced by viruses.

Therefore, viral sepsis should be considered when an acute viral infection causes potentially reversible, life-threatening organ dysfunction while excluding cases of chronic and latent infections that do not involve organ dysfunction and those caused by other pathogens.

### Diagnosis

The diagnostic approach to viral sepsis requires a synergistic integration of clinical assessments and laboratory evaluations. Clinically, a thorough patient assessment should include a detailed history of recent travel and potential exposure risks, in conjunction with common indicators of systemic inflammatory response syndrome.

Laboratory examinations, such as blood cultures, are essential for excluding bacterial etiologies. The emergence of viral-specific polymerase chain reaction (PCR) techniques enables the detection of viral nucleic acids [10]. Moreover, advanced molecular tools, including metagenomic next-generation sequencing [13], high-throughput sequencing, nanopore sequencing, and the CRISPR-based Fast Integrated Nuclease Detection in Tandem (FIND-IT), have demonstrated performance comparable to Centers for Disease Control and Prevention-recommended RT-qPCR assays, significantly enhancing our diagnostic capabilities [14–16].

**Fig. 2:**
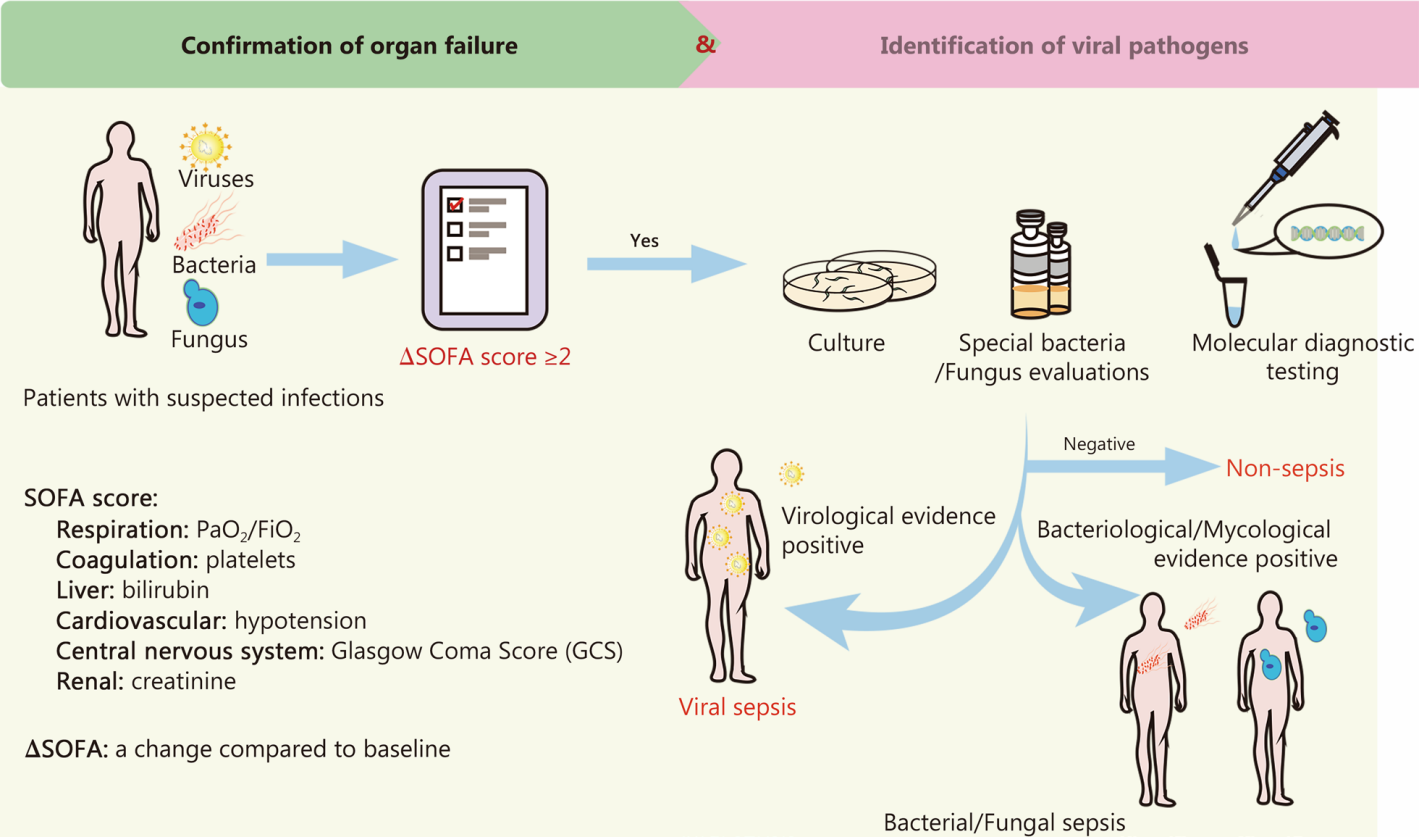
Diagnostic framework for viral sepsis. In cases where a patient exhibits infection markers or there is suspicion of an infection, and concurrently has a SOFA score ≥ 2 compared to baseline, it is imperative to exclude bacterial, parasitic, and fungal sources. If viral antigen or nucleic acid assays yield positive results, especially when supported by relevant epidemiological findings, the clinical presentation suggests a diagnosis of viral sepsis. Molecular diagnostic testing methods include viral-specific PCR, high-throughput sequencing, nanopore sequencing, and CRISPR-based FIND-IT. FIND-IT Fast Integrated Nuclease Detection in Tandem, PCR polymerase chain reaction, CRISPR clustered regularly interspaced short palindromic repeats, SOFA Sequential Organ Failure Assessment, PaO_2_/FiO_2_ the ratio of arterial oxygen partial pressure to fractional inspired oxygen

Viral serology is also instrumental in assessing the host’s immune response and is essential for differentiating between current and past viral infections. Other significant evaluations encompass biomarker analysis, imaging studies, SOFA score assessment, and a thorough differential diagnosis. In summary, when acutely ill patients present with unexplained organ dysfunction, indicated by a SOFA score of ≥ 2 from baseline and in the absence of evident bacterial, fungal, or parasitic sources, there should be a heightened suspicion of viral sepsis. These diagnostic evaluations are depicted in Fig. 2.

## Predominant pathogens: epidemiological profiles and clinical manifestations

Nearly 20 viruses are associated with multi-organ failure, a critical feature of sepsis, and exhibit specific geographic, seasonal, and endemic distributions (Table 1) [17–38].

**Table 1:** Common pathogens of viral sepsis

Common pathogen	Criteria for sepsis	Geographical distribution	Incidence and mortality	References
RSV	SOFA score ≥ 2; Negative bacterial culture; Positive virological evidence, including antigen test or viral-specific PCR, high-throughput sequencing, nanopore sequencing, and CRISPR-based FIND-IT; Combining clinical manifestations and imaging	Temperate areas and tropical regions	Incidence: affects all age groups. In high-income countries, around 5.2 million cases of RSV-related respiratory infections annually, with approximately 302,000–720,000 hospitalizations among adults aged 60 and older Case-fatality rate: among adults aged 60 and older, 16,000–67,000 deaths annually. In severe cases, the fatality rate has approached 30% in recent years. The rates may be even higher in resource-limited settings	[17, 18]
Influenza virus (e.g., H1N1, H7N9)		Global	WHO estimates 3–5 million cases of severe illness and 290,000–650,000 respiratory deaths occur annually	[19, 20]
MERS-CoV		Global	Incidence: from 2012 to 1 February 2024, over 2600 laboratory-confirmed cases of MERS have been reported to WHO; Case-fatality rate: approximately 36%	[21, 22]
SARS-CoV-2		Global	The disease caused by SARS-CoV-2 is an ongoing global health threat, as of 23 June 2024, there have been 775,678,432 reported COVID-19 cases and 7,052,472 deaths worldwide	[23]
Dengue virus		Americas; Southeast Asia; Western Pacific	Incidence: nearly half of the global population is at risk of dengue, with an estimated 100–400 million infections annually (WHO, n.d.) Mortality: from 1990 to 2017, age-standardized mortality increased in most countries, reaching approximately 30–40 per 100,000 people. In 2024, as of 30 April, 3.4 million confirmed cases, over 16,000 severe cases, and more than 3000 deaths have been reported to WHO	[24–26]
Dabie bandavirus		Asian countries (China; Korea; Japan; Vietnam; Myanmar; Thailand)	Incidence: the incidence of SFTS is on the rise, with over 20,000 cases reported by the end of 2021 in China, Korea, and Japan Case-fatality rate: the case-fatality rate ranges from 5.3 to 35%, depending on different virus genotypes	[27–29]
EBOV		West Africa	Incidence: by 2020, approximately 33,604 human EBOV infections had been recorded Mortality: The average case fatality rate is approximately 50%, with rates ranging from 25 to 90% in previous outbreaks, depending on conditions and the response	[30–33]
Hantavirus		Global	Incidence: over 200,000 cases are reported annually, including approximately 13,000 cases in China, 300–600 in South Korea, 3000 in the European Union, and 7000 in Russia Case-fatality rate: range from less than 1 to 44.5%, depending on the infecting virus	[34–36]
Lassa virus		West Africa; Benin; Ghana; Togo	Incidence: an estimated 500,000 to 897,700 cases of LASV occur annually in West Africa Mortality: the overall case-fatality rate is approximately 1%, with an observed rate of 15% among hospitalized patients with severe LASV infections (WHO, n.d.)	[37, 38]

*RSV* respiratory syncytial virus, *SOFA* Sequential Organ Failure Assessment, *SFTS* severe fever with thrombocytopenia syndrome, *MERS-CoV* Middle East respiratory syndrome coronavirus, *SARS-CoV-2* severe acute respiratory syndrome coronaviru*s* 2, *PCR* polymerase chain reaction, *CRISPR* clustered regularly interspaced short palindromic repeats, *FIND-IT* Fast Integrated Nuclease Detection in Tandem, *WHO* World Health Organization, *COVID-19* coronavirus disease 2019, *EBOV* Ebola virus, *LASV* Lassa virus, n.d. no date

### Respiratory viruses

Respiratory viruses, including rhinovirus, adenovirus, respiratory syncytial virus (RSV), influenza viruses, parainfluenza, and coronavirus, frequently lead to severe pneumonia (Table 1) [19–22]. Over 60% of such severe cases may present with sepsis [39, 40].

#### RSV

RSV affects individuals across all age groups and is increasingly recognized as a significant cause of acute and often severe respiratory illness, particularly in children under 5 years old and adults aged 60 and older, where hospitalization and mortality rates can be considerable [17, 41]. In high-income countries, RSV accounts for 4–12% of acute respiratory infection hospitalizations among adults aged 60 and older. National surveillance data from Brazil indicates a steady increase in RSV-related severe acute respiratory illness within this age group in recent years, with fatality rates exceeding 30% [17].

#### Influenza viruses

Influenza affects 5–10% of adults annually and is responsible for 3–5 million cases of severe illness and up to 650,000 deaths every year [19]. The influenza virus can lead to severe complications, including acute respiratory distress syndrome (ARDS), myocarditis, encephalopathy, and sepsis [42–44]. Avian influenza A (H1N1) and influenza A (H7N9), which cause community-acquired viral pneumonia, often result in multi-organ injury [45]. Notably, 38.5% of H7N9 cases may progress to septic shock [45], while the 2009 H1N1 pandemic significantly increases the risk of septic shock [46]. Similarly, avian influenza A (H5N1) frequently leads to multi-organ failure [47].

#### Coronaviruses

Lethal human coronaviruses include severe acute respiratory syndrome coronavirus (SARS-CoV), Middle East respiratory syndrome coronavirus (MERS-CoV), and SARS-CoV-2 [48]. SARS-CoV infections present sepsis-like symptoms such as fever, shortness of breath, and ARDS [49]. Approximately 78% of COVID-19 patients in Intensive Care Units (ICUs) meet the Sepsis-3.0 criteria [50, 51]. Research from Wuhan indicates that nearly all fatalities associated with SARS-CoV-2 involved sepsis, with 59 and 20% progressing to sepsis and septic shock, respectively [52]. MERS-CoV, primarily transmitted locally in the Middle East and reported in 27 countries by January 2020, has a mortality rate of 35%, often leading to death through septic shock, renal failure, and coagulative disorders [48].

### Dengue virus (DENV)

DENV, a mosquito-borne pathogen endemic to over 100 countries, predominantly affects the Americas, Southeast Asia, and the Western Pacific (Table 1) [24–26]. While many cases present as mild dengue fever with flu-like symptoms, severe dengue typically manifests with high fever, intense headaches, low white blood cell counts, and significant bleeding [53]. Dengue-related pulmonary complications, such as edema and alveolar hemorrhage, can mimic septic presentations [20]. Consequently, dengue is a prevalent cause of sepsis and severe sepsis among tropical infectious diseases in Southeast Asia. A study conducted in Thailand found that approximately 14% of sepsis patients tested positive for dengue via PCR assays of serum samples [42, 54]. Additionally, thrombocytopenia is a common and serious symptom that ranks among the most prevalent manifestations and significantly contributes to the severity of the disease.

### Dabie bandavirus (DBV)

DBV, a novel phlebovirus belonging to the Bunyaviridae family that causes severe fever with thrombocytopenia syndrome (SFTS), was first identified in rural areas of China in 2009 and has since been reported in East Asia and potentially the USA, highlighting its growing public health concern (Table 1) [27–29]. Primarily transmitted through tick bites, DBV has an incubation period of 6–14 d, with symptoms ranging from mild fever to severe, potentially fatal conditions. Severe cases of SFTS exhibit systemic, sepsis-like symptoms including hemorrhagic and neurological complications, thrombocytopenia, and disseminated intravascular coagulation (DIC), often leading to multi-organ failure characterized by acute kidney and liver impairments [55].

### Ebola viruses (EBOV)

EBOV, members of the Filoviridae family, cause Ebola virus disease (EVD), a severe and often fatal illness in humans (Table 1) [32]. First identified near the Ebola River, there have been at least 17 EVD outbreaks reported across Gabon, Guinea, and the Republic of the Congo [18]. EVD initially presents with fever and gastrointestinal symptoms, progressing to septic-like reactions and multi-organ damage. Clinical manifestations include hypotension, rhabdomyolysis, DIC, acute renal failure, hepatic failure, and central nervous system (CNS) complications [30, 56]. The average mortality rate for EVD is around 50% [30]. A critical factor in reducing Ebola mortality rates has been the successful deployment of vaccines, which hold promise for shaping future epidemic prevention strategies.

### Hantaviruses

Hantaviruses, classified within the Bunyavirales order, are increasingly recognized as significant public health concerns due to rodent transmission (Table 1) [34]. These viruses cause severe diseases such as hemorrhagic fever with renal syndrome (HFRS), characterized by acute kidney injury (AKI) and renal failure, and hantavirus pulmonary syndrome (HPS), which can progress to ARDS [34]. These conditions frequently lead to shock and elevated mortality rates. A multinational study identifying the causes of sepsis in Southeast Asia revealed hantaviruses as leading emerging pathogens [57]. With a mortality rate of approximately 12% for HFRS and significantly higher at 35–50% for HPS, focused research and surveillance of hantaviruses are essential [58].

### Lassa virus (LASV)

LASV, an enveloped RNA virus belonging to the Arenaviridae family, is primarily transmitted by the Mastomys natalensis rodent (Table 1) [38]. Approximately 20% of those infected develop severe disease, with the viruses impacting multiple organs such as the liver, spleen, and kidneys [21]. Research using a cynomolgus monkey model has shed light on LASV’s pathogenesis, indicating multi-organ failure similar to septic-shock syndrome [59]. Case fatality rates vary by region, with 10–20% of hospitalized Lassa fever patients succumbing to the disease [60, 61].

## Organ-specific dysfunctions in viral sepsis

**Fig. 3:**
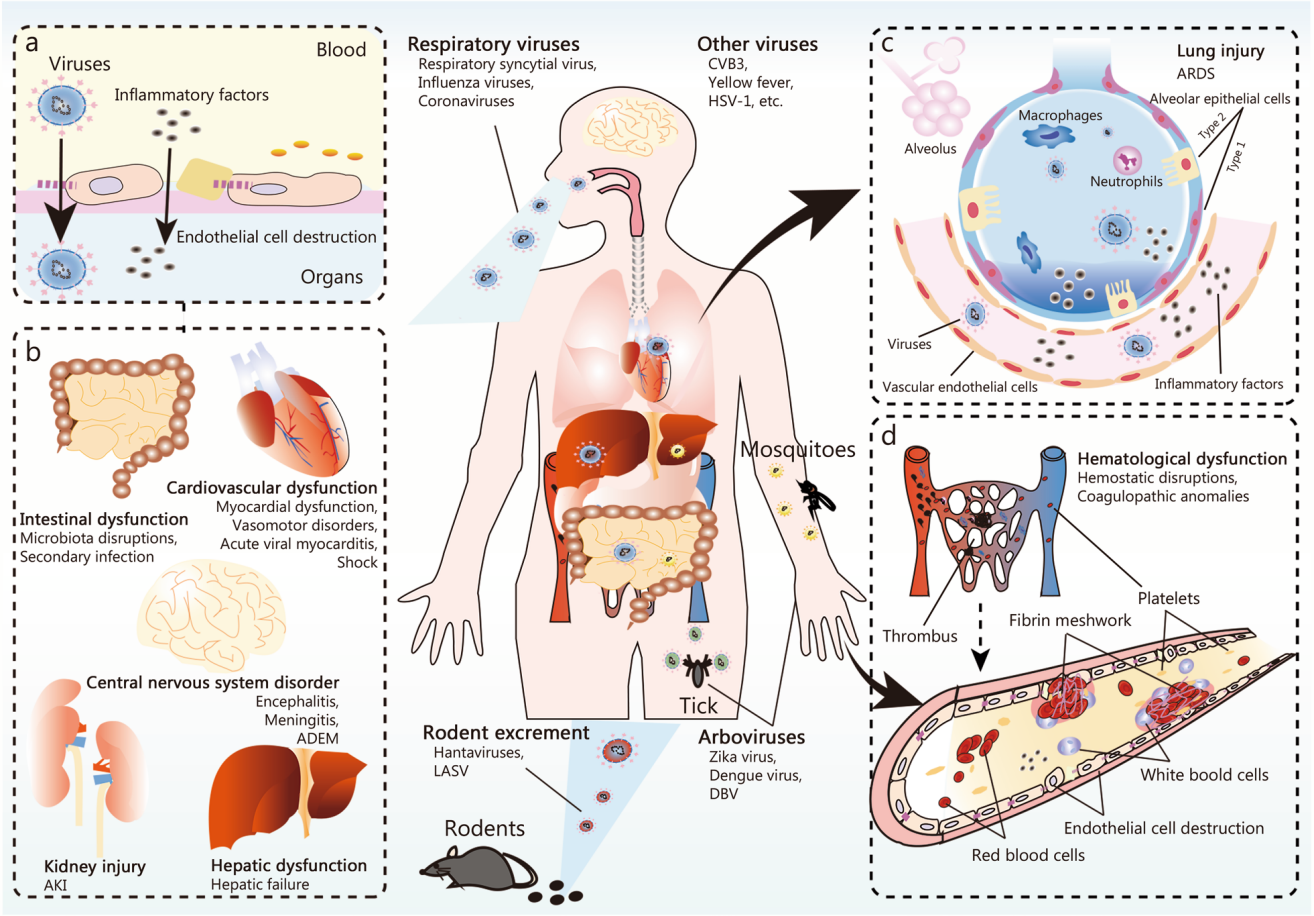
Viral sepsis pathogenesis and targeted organ vulnerability. The predominant etiological agents in viral sepsis are respiratory and vector-borne viruses. Respiratory viruses primarily transmit via the respiratory system, while vector-borne counterparts, such as tick-transmitted viruses, rely on insect vectors for transmission. Hantavirus and DBV spread through contact with rodents and exposure to their excreta. **a** Upon encountering these viruses, hosts often experience targeted cellular infections, and weaken the endothelial barrier in the vasculature. **b** Specifically, respiratory viruses penetrate alveolar epithelial cells or alveolar immune cells, multiplying within them. After inducing cellular damage, these pathogens migrate into the bloodstream, jeopardizing organs like such as liver, heart, kidneys, and intestines. **c** Conversely, arboviruses access the host circulatory system through skin contact or insect bites, and primarily target cells like platelets and fibrin meshwork. **d** The above mechanisms eventually induce coagulation disorders, manifesting symptoms reminiscent of hemorrhagic fever. Severe cases may result in extensive organ damage. Moreover, certain viruses, such as HSV, preferentially affect the nervous system, whereas others target the intestinal lining, including noroviruses and rotaviruses. The inherent cytotoxic effects of these viruses combined with host immune defenses heighten the potential for systemic organ damage that may culminate in multi-organ dysfunction. DBV Dabie bandavirus, HSV herpes simplex virus, ADEM acute disseminated encephalomyelitis, AKI acute kidney injury, CVB3 coxsackievirus B3, LASV Lassa virus, ARDS acute respiratory distress syndrome

The term “sepsis” has not been consistently applied to describe organ dysfunction resulting from viral infections [62]; however, the intrinsic cytotoxicity of the virus and the host’s immune responses contribute to organ damage and various complications (Fig. 3). Additionally, the number of organ failures is significantly correlated with increased mortality.

### Cardiovascular dysfunction and acute myocardial injury

Cardiovascular disturbances, including elevated troponin levels, rhythm abnormalities, myocardial dysfunctions, and vasomotor disorders, are grouped under septic cardiomyopathy, primarily triggered by bacterial systemic inflammation. This condition is generally reversible with timely intervention [63, 64]. Similarly, viral infections such as influenza [65], SARS-CoV-2 [66], dengue fever [67], hantavirus [68], and Ebola [69] can elicit systemic inflammatory responses that lead to virus-induced septic cardiomyopathy, which also typically responds well to appropriate management.

However, viruses such as coxsackievirus B3 (CVB3), adenovirus, parvovirus B19, MERS-CoV, SARS-CoV, and particularly SARS-CoV-2 exhibit a significant propensity for myocardial invasion, resulting in acute viral myocarditis [66]. While recovery is frequently observed, unresolved myocarditis may progress to chronic heart failure [70]. Myocardial complications associated with viral infections generally present poor prognostic outcomes. In one study involving hospitalized COVID-19 patients, myocardial injury was correlated with an in-hospital mortality rate of 51% [71].

### Lung injury and ARDS

In the context of viral sepsis, the heightened immune response, while essential for viral clearance, damages the vascular and alveolar structures of the lung, leading to lung injury and ARDS, which presents as dyspnea, hypoxemia, and respiratory distress [72]. Viruses such as specific influenza subtypes (e.g., H1N1, H3N2) and coronaviruses (SARS-CoV, MERS-CoV, and SARS-CoV-2) are prominent etiological agents for ARDS [73, 74]. Hantaviruses can lead to HPS, a severe respiratory condition potentially progressing to ARDS. Although less common, pathogens such as RSV, adenoviruses, human metapneumovirus, parainfluenza viruses, and rhinoviruses can also trigger ARDS, particularly in susceptible populations like children [75].

During the 2009 H1N1 pandemic, ARDS affected 18–33% of patients admitted to ICU, with mortality rates ranging from 25 to 40% [76]. The SARS-CoV outbreak resulted in ARDS in approximately 20–30% of hospitalized patients, with a mortality rate nearing 50% [77]. In the COVID-19 pandemic, ARDS-related fatalities varied between 26 and 44% [77–80].

### Kidney injury and AKI

AKI in the context of viral sepsis is characterized by a sudden decline in renal function, leading to disruptions in fluid and electrolyte balance. Its etiology is multifactorial, involving direct viral invasion of renal cells, immune-mediated injuries, hemodynamic changes, and adverse effects from antiviral therapies. Viruses such as influenza, hantavirus, dengue fever, adenoviruses, polyomaviruses, and particularly SARS-CoV-2, are implicated in the pathogenesis of AKI [81].

In H1N1, avian influenza, and SARS-CoV-2 infections, AKI results from direct renal assault, rhabdomyolysis, cytokine storms, and systemic effects [81]. Dengue-induced AKI may arise from systemic inflammatory response syndrome, hypotension, or direct renal invasion [82]. Conversely, hantaviral infections can lead to HFRS, resulting in kidney injury [83].

The reported incidence of AKI in H1N1 influenza ranged from 34 to 53%, with an in-hospital mortality rate of approximately 36% [84]. Up to 40–50% of patients with MERS-CoV infection may develop acute renal failure [85, 86]. During the COVID-19 pandemic, the occurrence of AKI varied between 0.5 and 46% [87], with higher rates observed in ICU settings, and corresponding mortality rates around 50%, ranging from 7% to as high as 100% [81, 88, 89].

### Haematological dysfunction

Hematological dysfunction in viral sepsis arises from direct viral effects on the hematopoietic system and immune-mediated damage. This dysfunction typically manifests as thrombocytopenia, coagulopathies, and DIC [90]. Thrombocytopenia is frequently observed in severe viral sepsis [91], with viruses such as influenza, DENV, hantavirus, and DBV causing severe thrombocytopenia and coagulation disorders [92, 93]. Notably, 40–50% of SARS-CoV-2 cases exhibit thrombocytopenia, with in-hospital mortality rates ranging from 17.5 to 92.1% [94, 95]. DIC is characterized by systemic microvascular thromboses that impair organ perfusion, leading to multi-organ dysfunction such as AKI, ARDS, and cerebral stroke while predicting mortality [96]. The EBOV often induces DIC, resulting in hemorrhagic fever and systemic organ failure [97].

### CNS disorder

The CNS is particularly susceptible to viral infections that can lead, either directly or indirectly, to various neurological disorders [98]. Directly, viruses such as herpes simplex virus types 1 and 2 (HSV-1, HSV-2), arboviruses including Zika virus (ZIKV) and West Nile virus (WNV), and SARS-CoV-2 target CNS components such as neurons and vascular endothelial cells (ECs), resulting in conditions including encephalitis, meningitis, stroke, and cognitive disruptions [98, 99]. Indirectly, viruses like DENV, influenza virus, and SARS-CoV-2 may cause CNS complications via mechanisms such as cytokine release, CNS-antibody cross-reactions, cytotoxic T cell activation, and disruption of the blood-brain barrier (BBB). Systemic factors such as hypoxia and multi-organ failure can further exacerbate these conditions [98]. Autopsies of COVID-19 patients have shown the presence of SARS-CoV-2 in cortical neurons accompanied by immune cell infiltration [99].

### Hepatic dysfunction

Hepatic dysfunction in viral infections is influenced by both viral pathogenesis and host factors, including age, immune status, and pre-existing liver conditions [100]. While hepatitis viruses are primary contributors to liver impairment, other systemic viruses such as EBV, CMV, DENV, yellow fever virus, DBV, ZIKV, EBOV, and SARS-CoV-2 also play a significant role [101]. These viruses can directly target hepatocytes and Kupffer cells, leading to apoptosis, necrosis, or pyroptosis. Additionally, liver injury in the context of viral sepsis may result from aggressive immune responses or hypoxic damage [100–102]. Hepatic impairment has been observed in up to 60% of SARS cases [103] and between 15 and 50% of COVID-19 cases [102]. Similar findings have been reported in MERS-CoV infections [104].

### Intestinal dysfunction

Intestinal dysfunction in viral sepsis stems from direct viral damage to epithelial cells, as well as indirect effects such as immune-mediated injuries and disruptions in gut microbiota [105]. Viral cytopathic effects increase intestinal permeability, leading to fluid loss-induced hypotensive shock and elevated risk of secondary bacterial infections [105]. Concurrently, systemic inflammation exacerbates mucosal damage in the gut and promotes bacterial translocation into the bloodstream, thereby amplifying the inflammatory cycle. Disturbances in the gut microbiota significantly worsen intestinal dysfunction and contribute to the progression of sepsis. Viruses including noroviruses, rotaviruses, enteroviruses, HSV, CMV, influenza virus, and SARS-CoV-2 are recognized contributors to these pathologies [106].

## Mechanism of organ dysfunction in virus infection-induced sepsis

Organ dysfunction induced by viruses may exhibit comparable clinical manifestations, yet it entails distinct signaling pathways.

### Activation of signaling pathways and viral immune evasion strategies

Upon viral infection of the host, PRRs such as Toll-like receptors (TLRs), nucleotide-binding and oligomerization domain (NOD)-like receptors (NLRs), and intracellular sensors including RIG-I, absent in melanoma 2 (AIM2) and cGAS recognize the viral components. This recognition triggers a signaling cascade that primarily leads to the secretion of type I interferons (IFNs) to combat the virus [107]. In cases of viral sepsis, dysfunctional signaling may impede viral clearance, facilitate viral evasion, and exacerbate tissue injury.

#### TLRs signaling pathway unmethylated cytosine-phosphate-guanine

TLRs consist of 10 members. Surface TLRs (TLR1, TLR2, TLR4, TLR5, TLR6, and TLR10) detect viral coat proteins [108], while endolysosomal TLRs (TLR3, TLR7, TLR8, and TLR9) recognize specific viral nucleic acids [109]. Specifically, TLR3 identifies double-stranded RNA, such as that from DENV [110]. Whereas both TLR7 and TLR8 recognize single-stranded RNA from viruses including influenza viruses, coronaviruses, and flaviviruses [111]. Additionally, TLR9 detects unmethylated cytosine-phosphate-guanine DNA motifs [109]. Upon ligand binding, these receptors initiate signaling cascades through myeloid differentiation factor 88 or the Toll/interleukin-1 receptor/resistance protein (TIR) domain-containing adapter inducing IFN-β, which are crucial for targeting viral infections. However, some viruses, including vaccinia virus, MERS-CoV, and SARS-CoV-2, manipulate the signaling pathways of TLRs to enhance their replication by deactivating the TIR domain-containing adaptor inducing interferon-β adaptor, inhibiting inhibitor kappa B kinase alpha and inhibitor kappa B kinase beta kinases, and upregulating suppressor of cytokine signaling proteins. This manipulation exacerbates viral evasion mechanisms and adverse outcomes [112–114].

#### NLRs signaling pathway

NLRs are categorized into subfamilies based on their N-terminal domains, which include acidic transactivation (NLRA), baculoviral inhibitory repeat-like (NLRB), caspase activation and recruitment (NLRC), and pyrin (NLRP) [115]. The NLRA exclusively comprises the major histocompatibility complex (MHC) class II transactivator, while NLRB suppresses apoptosis by inhibiting caspases-3, -7, and -9. The NLRC subfamily comprises NLRC1–5 and NLRX, characterized by a caspase activation and recruitment domain for facilitating caspase recruitment [116, 117].

Specifically, NLRC2 (NOD2) detects cytoplasmic viral components, such as single-stranded RNA from RSV, vesicular stomatitis virus, influenza A virus (IAV), parainfluenza virus 3, MERS-CoV, and SARS-CoV-2, triggering the production of IFN-β via the mitochondrial antiviral signaling (MAVS) pathway, essential for antiviral defense [118–120]. Other members of the NLRC family such as NLRX1, NLRC5, and NLRC3, mainly modulate the IFN pathways [121]. For example, NLRC3 binds to HSV dsDNA, enhancing type I IFN production by limiting its interaction with STING and TRAF family member-associated NF-κB activator (TANK)-binding kinase 1 (TBK1) [122]. Additionally, NLRC5 inhibits viral infection by blocking RIG-I and anti-melanoma differentiation-associated protein 5 (MDA5) activation while regulating MHC class I expression.

The NLRP subfamily, characterized by pyrin domains, forms inflammasomes. The NLRP3 inflammasome, activated by viral RNA and proteins, is implicated in lung injuries and ARDS during infections with MERS-CoV, SARS-CoV-1, and SARS-CoV-2 [123], as well as causing significant renal damage during ZIKV infection [124]. NLRP1 is activated by double-stranded RNA, while NLRP6 collaborates with RNA helicase DEAH-box helicase 15 and MAVS to enhance antiviral defenses. Notably, deficiency of NLRP6 in mice is associated with increased gastrointestinal viral loads [121].

#### RIG-I-like receptors signaling pathway

RLRs, including RIG-I, MDA5, and laboratory of genetics and physiology 2 (LGP2), play a critical role in the antiviral immune response [125]. Both RIG-I and MDA5 contain caspase activation and recruitment domains, whereas LGP2 lacks these domains and primarily regulates the activities of RIG-I and MDA5. These receptors, using a central helicase domain, recognize the 5’-triphosphate ends of RNA from various viruses, including Herpesviridae (e.g., HSV, EBV), vaccinia virus, Flaviviridae (e.g., WNV, HCV, ZIKV), coronaviridae (e.g., SARS), Filoviridae (e.g., EBOV, Marburg virus), HIV, and HBV. Specifically, MDA5 recognizes adenovirus and IAV [126].

Upon the detection of RNA, RLRs initiate a signaling cascade through interactions with MAVS, which subsequently activates TBK1. TBK1 then phosphorylates interferon regulatory factor (IRF)3 and IRF7, leading to the production of type I IFN-α/-β and pro-inflammatory cytokines [126]. This pathway is crucial in SARS-CoV-2 infection and is associated with increased disease severity [125]. Additionally, RIG-I interacts with components of the inflammasome such as apoptosis-associated speck-like protein containing a caspase-recruitment domain (ASC) and caspase-1, thereby promoting the formation of virus-specific inflammasomes during viral infections including vesicular stomatitis virus and IAV [125].

#### cGAS-STING signaling pathway

The cGAS-STING pathway serves as a broad-spectrum cytosolic sensor that detect nucleotides from various viruses, including DNA viruses (e.g., vaccinia virus, HSV-1, Kaposi’s sarcoma-associated herpesvirus, adenoviruses), retroviruses (e.g., HIV-1, HIV-2), and RNA viruses (e.g., DENV, norovirus, IAV, encephalomyocarditis virus, SARS-CoV-2) [127]. Upon the detection of DNA, cGAS synthesizes 2’-3’-cyclic-GMP-AMP, which subsequently activates STING. STING then translocates to the Golgi, facilitating the transcription of antiviral genes via TBK1-IRF3 signaling and NF-κB activation [128].

Furthermore, STING directly responds to RNA viruses by interacting with viral proteins. For example, the open reading frame (ORF) 9b protein of SARS-CoV-2 impairs IRF3 phosphorylation [128], while the nonstructural protein 1 (NS1) protein of ZIKV reduces IFN-I responses [129]. The activation of the STING pathway is linked to pulmonary inflammation in COVID-19 as well as increased intestinal inflammation in cases of abdominal sepsis [130].

### Cellular stress responses and metabolic disorders

Viruses can also manipulate host cellular processes, including endoplasmic reticulum (ER) stress, enhanced glycolysis, and lipid biosynthesis, thereby exacerbating inflammation and contributing to further organ damage.

#### ER stress

ER stress and the unfolded protein response are critical for restoring cellular homeostasis during viral infections. Persistent viral-induced ER stress exacerbates inflammation and organ damage through pathways such as protein kinase R-like ER kinase (PERK)-eukaryotic initiation factor 2 alpha (eIF2α)-activating transcription factor 4 (ATF4) and C/EBP homologous protein (CHOP) [131, 132]. The ORF8 protein and accessory protein 3a of SARS-CoV-2 amplify ER stress by activating ATF6, inositol-requiring enzyme 1, ATF4, and CHOP [133]. This stress can lead to conditions such as ARDS and other organ complications. Similar roles of ER stress in viral pathology have been observed in Crimean-Congo hemorrhagic fever virus [133], influenza B viruses, WNV, CVB3, and HIV.

#### Mitochondrial dysfunction and intracellular metabolic disorders

Viruses exploit mitochondria to enhance replication and evade immune responses. Certain viruses, such as HBV, HCV, and EBV, promote mitochondrial fission [134], while SARS-CoV and DENV facilitate fusion [135]. This disruption of mitochondrial function can lead to cell death and organ failure [136]. Additionally, virus-induced ER stress may further compromise mitochondrial function by altering calcium homeostasis [137].

Furthermore, mitochondria play a critical role in energy production through pathways such as the tricarboxylic acid cycle, fatty acid oxidation, the electron transport chain, and oxidative phosphorylation (OXPHOS). Viral infections can disrupt these pathways. For example, human cytomegalovirus affects both the tricarboxylic acid cycle and the electron transport chain, leading to an increase in reactive oxygen species (ROS) and subsequent mitochondrial damage. This damage results in the release of mitochondrial DNA, which acts as mitochondrial damage-associated molecular patterns (mtDAMPs), intensifying systemic inflammation via the cGAS-STING pathway. Elevated levels of mtDAMPs are associated with severe outcomes in bacterial sepsis [138, 139].

Viral infections can also shift metabolism from OXPHOS to glycolysis [140], promoting viral replication and leading to lactate accumulation and mitochondrial dysfunction [141]. This dysfunction may result in cardiac issues, weakened immune defenses, particularly in diseases such as dengue fever [142], and neuroinflammation in neurons that rely on OXPHOS.

Pathological lipid accumulation due to viral replication can exacerbate ARDS in SARS-CoV-2-infected hosts [141], leading to cardiac dysfunction, and compromising BBB integrity. Disruption in amino acid metabolism can also affect neurotransmitter balance and BBB integrity, impacting CNS diseases, as evidenced by alterations in tryptophan metabolism [141].

### Cell death pathways activation

Cell death mechanisms such as apoptosis, necroptosis, pyroptosis, ferroptosis, and PANoptosis, are conserved strategies employed to restrict viral replication. However, their excessive activation can result in lymphocyte depletion, immunosuppression, tissue injury, and organ failure, leading to adverse outcomes [143].

#### Apoptosis

Viruses often manipulate the host’s apoptotic machinery through both extrinsic and intrinsic pathways. Disruptions of these pathways by viruses induce mitochondrial membrane permeabilization and cytochrome-c release, triggering the formation of the apoptosome and activating the caspase cascade, ultimately leading to apoptosis. Additionally, viruses can amplify intrinsic apoptosis via interferon-stimulated genes, RLRs, and cGAS [109]. Autopsy results from COVID-19 patients reveal increased T cell apoptosis, mirroring patterns observed in infections caused by IAV and DENV [144]. In bacterial sepsis, uncontrolled apoptosis significantly depletes immune cells such as B cells and CD4^+^ T lymphocytes, thereby weakening immune defenses [145] and increasing vulnerability to secondary infections, which often correlates with poor prognoses.

#### Necroptosis

When viruses inhibit apoptosis, necroptosis serves as an alternative cell death pathway. Necroptosis can be triggered by death receptors such as TNF receptor, factor-related apoptosis (FAS/CD95), TLR3, TLR4, and Z-DNA binding protein 1 (ZBP1). Upon activation, ZBP1 interacts with receptor-interacting protein kinase 3 (RIPK3), facilitating the phosphorylation of mixed lineage kinase domain-like protein. This process results in mixed lineage kinase domain-like protein oligomerization, cell membrane rupture, and the release of high mobility group box 1 protein, cytokines, and histones, leading to significant inflammation and exacerbating organ damage.

Several viruses, including IAV, SARS-CoV-2, HSV-1, CVB3, and RSV, can induce necroptosis. IAV activates ZBP1, leading to lung epithelial cell death and an increased risk of ARDS [146]. SARS-CoV-2 infection induces the formation of Z-RNA, thereby activating the ZBP1-RIPK3 pathway [147]. Elevated plasma levels of RIPK3 in sepsis patients are linked to a higher incidence of ARDS and AKI. In neural tissues, HSV-1 utilizes infected cell protein 6, the large subunit of ribonucleotide reductase, to suppress caspase-8 [148], initiating necroptosis and leading to encephalitis. CVB3 induces necroptosis in cardiac cells, resulting in myocarditis.

#### Pyroptosis

Similar to necroptosis, pyroptosis releases intracellular materials, including IL-1β, IL-18, and high mobility group box 1, leading to tissue injury [149]. Pyroptosis relies on the formation of inflammasome, involving NLRP family members (NLRP1 and NLRP3) as well as hematopoietic interferon-inducible nuclear proteins with a 200-amino acid repeat (HIN200)-containing proteins such as AIM2 and interferon-inducible protein 16. AIM2 and interferon-inducible protein 16 are DNA receptors that induce inflammasome formation and promote interferon production, respectively [150]. NLRP1 and NLRP3 detect viral activities and trigger inflammasome assembly. The SARS-CoV-2 N (nucleocapsid) protein facilitates the assembly of the NLRP3 inflammasome, leading to IL-1β production and lung injury in mice [151, 152]. IAV may increase the risk of lung injury by activating the NLRP3 inflammasome through galectin-3 in respiratory epithelial cells [153]. CVB3 and ZIKV activate pyroptosis in cardiomyocytes and neural cells, causing myocarditis and potential encephalitis, respectively. DENV induces pyroptosis via the NLRP3 inflammasome by directly engaging caspase-4, thereby amplifying organ damage, particularly in the liver and heart during acute phases [148].

#### Ferroptosis

Ferroptosis is a non-apoptotic programmed cell death characterized by the accumulation of iron-dependent lipid peroxides [149, 154]. Viruses frequently exploit ferroptosis to facilitate viral transmission and cause host organ damage. Viruses such as CVB3, IAV, mouse mammary tumor virus, and canine parvovirus, bind to transferrin receptor 1 to enter cells, leading to iron accumulation and subsequent ferroptosis. EBV indirectly influences ferroptosis by promoting nuclear factor erythroid 2-related factor 2, which inhibits glutathione peroxidase 4 (GPX4), a key regulator of ferroptosis, while simian immunodeficiency virus, SARS-CoV-2, and Newcastle disease virus directly suppress GPX4 to induce ferroptosis. Additionally, coxsackievirus (CV)-A6, ZIKV, and IAV activate lipid peroxidation, while mouse hepatitis virus-A59 upregulates acyl-CoA synthetase long-chain family member 1, inducing ferroptosis through the NF-κB and TLR4 pathways [155]. In septic progression, decreased hepatic GPX4 and glutathione levels, along with increased indicators of ferroptosis such as malondialdehyde, lipid ROS, and Fe^2+^, are observed [156].

#### PANoptosis

PANoptosis is an inflammatory programmed cell death pathway that integrates features of pyroptosis, apoptosis, and necroptosis [157]. This process is primarily coordinated by the PANoptosome complex, which involves RIPK1, RIPK3, caspase-8, NLRP3, ASC, and FAS-associated protein with death domain (FADD) [157]. Upon viral infection, ZBP1 acts as an upstream sensor, assembling the ZBP1-PANoptosome with proteins such as RIPK3 and caspase-8, leading to lung injury in infections like IAV [158]. Elevated levels of ZBP1 have been observed in severe COVID-19 patients [130]. The AIM2-PANoptosome mediates PANoptosis during HSV-1 infections [158]. Moreover, cytokine release induced by viruses, especially those enhanced through interactions between TNF and IFN-γ, can amplify caspase-8/FADD-mediated PANoptosis, exacerbating lung injury [158].

#### Autophagy

Autophagy can degrade viral proteins or virions [159]. However, many viruses exploit this process to enhance replication, facilitate transmission, and cause host organ damage. Viruses such as mouse hepatitis virus, MERS-CoV, SARS-CoV, and SARS-CoV-2 induce double-membrane vesicles that serve as sites for replication and assist in exporting viral RNAs to the cytosol, leading to cytokine release and organ damage [160]. Some viruses inhibit the fusion of autophagosomes with lysosomes by targeting components such as soluble N-ethylmaleimide-sensitive factor attachment protein receptors, Ras-related protein in the brain (Rab) GTPases, tethering factors, or lysosomal functions. For instance, CVB3 protease 3C, human parainfluenza virus type 3 P protein, and Ebola viral protease target SNARE-binding protein synaptosomal-associated protein of 29 kD to inhibit autophagy flux. SARS-CoV-2 proteins ORF3a and ORF7a disrupt lysosomal fusion [161], while IAV M2 and DBV NSs proteins block autophagosome-lysosome fusion by interacting with Beclin1 [162]. Additionally, viruses such as ZIKV, HCV, WNV, and DENV exploit secretory autophagy for viral maturation, egress, and cell-to-cell spread, significantly enhancing their replication within the host [162–164].

### Cytokine and histones release

Cytokines and intracellular histones are well-established inflammatory molecules released by activated or dying cells, and their release can directly elevate the risk of tissue damage and organ failure [149].

#### Cytokine release

Viruses such as influenza, SARS-CoV, MERS-CoV, and SARS-CoV-2, induce a state of hyperinflammation characterized by elevated levels of IL-1β, IL-6, and IFN-γ. This condition disrupts the alveolar-capillary interface, resulting in pulmonary complications such as ARDS [165]. The cytokine storm involving IFN-α, C–C chemokine motif ligand 2, IL-6, and TNF, is often associated with mortality related to influenza [165] and heightens the risk of AKI and neuroinflammation. Similarly, DENV and EBOV exacerbate cytokine release, leading to increased vascular permeability in dengue hemorrhagic fever and fatal shock in EBOV [166].

#### Histones release

During viral infections, histones such as H1, H2A, H2B, H3, and H4 are released extracellularly through processes like NETosis, apoptosis, and necrosis, acting as DAMPs that bind to TLRs. This activation triggers the release of cytokines and chemokines, initiating inflammatory responses that recruit immune cells and result in organ damage. Extracellular histones also upregulate endothelial adhesion molecules such as E-selectin and intercellular adhesion molecule 1, facilitating leukemia cell adhesion and migration while contributing to endothelial and epithelial cell damage as well as platelet aggregation [167, 168]. In severe cases of COVID-19, elevated levels of circulating extracellular histones are linked to myocardial infarction, stroke, coagulopathy, and systemic hyperinflammation [169–171]. Citrullinated histone H3-positive neutrophils indicate thrombotic inflammation in COVID-19 [172]. Additionally, accumulation of extracellular histones along with pulmonary microvascular thrombosis has been observed in both influenza-infected mice and human cases [173].

### Immune cell activation and virus clearance

The innate immune system, consisting of neutrophils, macrophages, and dendritic cells, is the first line of defense that detects and responds to viral infections in tissues (Table 2) [174–183]. If the virus successfully evades this initial response, the adaptive immune system activates T cells to target infected cells and B cells to produce neutralizing antibodies. However, if the infection persists, it may lead to viremia and excessive activation of immune cells [184].

**Table 2:** The roles and mechanisms of pivotal immune cells in organ dysfunction triggered by virus-induced sepsis

Immune cell name	Type	Activation mechanisms	Functions in defensing viruses	Adverse outcome	Modulation by viruses	References
Neutrophil	Innate immune cell	Recognize PAMPs and DAMPs through PRRs and initiate signaling pathways including NF-κB; Elevated ROS promotes the translocation of neutrophil elastase to the nucleus, to induce neutrophil extracellular trap (NET) formation	Phagocytosis; Degranulation; NET release	Intensified inflammation; Tissue damage; Coagulation abnormalities; Multi-organ dysfunction	Viruses instigate NET production directly through PRRs on neutrophils or indirectly via cytokine release	[174, 175]
NK cell	Innate immune cell	Activating receptors such as NKG2D, NCRs, and CD16 identify compromised cells; Inhibitory receptors like KIRs and NKG2A ensure self-tolerance on healthy cells	Release cytolytic agents like perforin and granzymes; Release pro-inflammatory cytokines; Release chemokines to recruit immune cells and influence T and B cell dynamics	Cytokine storm; Organ dysfunction; Endothelial cell damage	Viruses disrupt the balance between activating receptors and inhibitory receptors, thereby amplifying NK cell activation	[176, 177]
B cell	Adaptive immune cell	Recognize viral antigens via BCR and trigger signaling cascades involving Src, and Syk kinase; Collaborate with CD4^+^ T cells through CD40-CD40L interaction	Produce specific antibodies and memory cells; Secret pro-inflammatory cytokines like IL-6, BAFF and APRIL; Release chemokines to attract immune cells	Cytokine storm; Endothelial structures damage; DIC; Organ injury	Sustained viral exposure leads to B cell exhaustion; Viruses increase the number of Bregs and lead to immunosuppression	[178–180]
T cell	Adaptive immune cell	Recognize peptide-MHC complexes presented by antigen-presenting cells; Co-stimulatory interactions amplify T cell activation; Intracellular signaling cascades including Src-family kinase and Zap70 kinase can activate transcription factors such as NFAT, AP-1, and NF-κB	CD8^+^ T cells directly target infected cells; CD4^+^ T cell bolster B cell activation, antibody generation and secret cytokine	Systemic inflammation; Organ damage; Microthrombosis	Chronic virus exposure predisposes T cells to exhaustion and leads to immunosuppression	[181–183]

*BCR* B cell receptor, *PAMPs* pathogen-associated molecular pattern molecules, *PRRs* pattern recognition receptors, *DAMPs* damage-associated molecular patterns, *NFAT* nuclear factor of activated T cells, *AP-1* activator protein-1, *DIC* disseminated intravascular coagulation, *NKG2D* natural killer group 2 member D, *NCRs* natural cytotoxicity receptors, *BAFF* B cell activating factor, *APRIL* a proliferation-inducing ligand, *NK* natural killer, *Src* steroid receptor coactivator

#### Neutrophil activation and neutrophil extracellular traps (NETs) formation

Upon viral infection, neutrophils engage in phagocytosis, degranulation, and the release of NETs to combat pathogens (Table 2) [174, 175]. These cells activate pro-inflammatory signaling pathways, thereby amplifying ROS production via the reduced nicotinamide adenine dinucleotide phosphate oxidase complex. Increased ROS levels promote the translocation of neutrophil elastase to the nucleus, where it collaborates with myeloperoxidase to induce chromatin decondensation and NET formation, a process known as NETosis [174]. NETs, composed of DNA fibers and histones, facilitate fibrin deposition, platelet capture, and thrombus stabilization [174]. Excessive NET formation in the lungs has been associated with ARDS and coagulation abnormalities in COVID-19 [175]. Elevated serum markers for NETs correlate with disease severity in influenza, dengue fever, and COVID-19. Remnants of NETs have been identified in various organs during post-mortem examinations of COVID-19 patients [175].

#### NK cell activation

NK cells are innate lymphocytes that play a crucial role in responding to acute viral infections [176]. They eliminate viruses and coordinate adaptive immunity through cytotoxic actions and cytokine release. Upon activation, NK cells secrete perforin and granzymes (Table 2) [175, 176], which destroy infected cells but may also damage adjacent healthy cells, leading to tissue injury. For instance, hantavirus infections can activate NK cells, resulting in damage to ECs and inducing thrombosis in the lungs, liver, and kidneys. Additionally, activated NK cells release pro-inflammatory cytokines such as IFN-γ, TNF, and granulocyte–macrophage colony-stimulating factor (GM-CSF) [177], which can trigger a potentially dangerous “cytokine storm” that compromises organ function. This phenomenon is observed in flavivirus and IAV infections, characterized by significant NK cell activity and cytokine production in the lungs [177]. In COVID-19, chemokines released by NK cells exacerbate inflammation by attracting additional immune cells, thereby affecting T and B cell dynamics.

#### B cell activation and exhaustion

B cells are essential at all stages of the immune response. They become activated upon recognizing viral antigens through their B cell receptor or via PRRs such as TLR7 and TLR9. Once activated, they differentiate into plasma cells that produce antibodies to neutralize and eliminate viral particles (Table 2) [177, 178]. However, excessive antibody production can lead to inflammatory tissue damage, including glomerulonephritis [185, 186]. In addition to producing antibodies, B cells secrete pro-inflammatory cytokines such as IL-6, B cell activating factor, and a proliferation-inducing ligand, which can damage endothelial integrity and impair organ function in severe viral infections like EBOV, influenza, and SARS-CoV-2 [178, 179]. This cytokine release, combined with B cell-derived antibodies, can disrupt coagulation processes, increasing the risk of DIC and contributing to severe outcomes such as ARDS [187].

Viral exposure can also result in B cell exhaustion or apoptosis. For example, IAV can induce B cell death, thereby compromising protective immunity. Furthermore, certain viruses promote the development of immunosuppressive regulatory B cells, leading to immune paralysis and secondary infections, ultimately exacerbating outcomes in sepsis [180].

#### T cell activation and exhaustion

T cells are pivotal components of adaptive immune responses and play a crucial role in viral elimination. Activated by antigen-presenting cells such as dendritic cells via MHC I or II molecules, T cells trigger intracellular signaling cascades through their T-cell receptors, involving steroid receptor coactivator-family kinases and Zap70 (Table 2) [181, 182]. Cytokines including IL-2, IL-6, IL-12, and IL-23 facilitate T cell activation, expansion, and maturation, thereby enabling their migration to sites of infection.

Activated CD4^+^ T cells facilitate B cell activation and antibody production, while CD8^+^ T cells specifically target virus-infected cells [182]. These processes are vital for controlling infections caused by viruses such as measles, CMV, HCV, and HIV. However, excessive T cell activation can result in tissue damage and exacerbate conditions such as COVID-19 and influenza by amplifying inflammatory responses and increasing vascular permeability, leading to complications like ARDS [182] and microthrombosis.

Persistent viral infections can result in T cell exhaustion. Severe cases, such as those involving SARS-CoV-2, frequently exhibit lymphopenia, which is associated with poor clinical outcomes (Table 2) [183]. Regulatory T cells can also suppress antiviral responses, thereby promoting viral persistence and further complicating the immune response [188].

### Immunosuppression in viral sepsis

Immunosuppression in viral sepsis compromises both the innate and adaptive immune systems, characterized by imbalanced cytokine profiles, lymphocyte apoptosis, and exhaustion, increased expression of inhibitory receptors such as programmed death-1 and cytotoxic T-lymphocyte antigen 4, elevated levels of myeloid-derived suppressor cells (MDSCs), and weakened pathogen defense. This state is typically evident in the bloodstream from the onset of infection through hospitalization [189], heightening susceptibility to secondary infections [42], prolonged illness, organ damage, and increased mortality rates [190].

Viruses such as CMV, EBOV, measles virus, influenza virus, and particularly SARS-CoV-2, are closely associated with this immunosuppressive condition [42]. In severe COVID-19 cases, there is a significant rise in MDSC levels in both blood and lung tissues [191]. Viruses like influenza induce immune cell apoptosis and disrupt cytokine balances, impairing the function of T cells, B cells, and NK cells, while increasing the presence of suppressive cells such as regulatory T cells and MDSCs [192].

### Non-immune component activation and virus clearance

Non-immune components such as ECs, platelets, and the complement systems are also primary targets of cytokines and intracellular materials. While their activation can facilitate virus clearance, it also triggers endothelial damage, and coagulation dysfunction, and increases the risk of tissue injury and organ failure.

#### EC activation

ECs are activated to limit pathogen spread [193], but excessive activation increases endothelial permeability perturbations, as evidenced by glycocalyx degradation and disruptions in junction proteins such as vascular endothelial-cadherin. Viruses like the DENV NS1 protein damage the glycocalyx, while hantaviruses destabilize endothelial integrity by inhibiting β3 integrins [193, 194]. SARS-CoV-2 further exacerbates these perturbations, leading to ARDS and myocardial ischemia [195]. In the gastrointestinal tract, these disruptions can lead to bacterial translocation, thereby increasing the risk of sepsis. Activated ECs also express elevated levels of adhesion molecules such as E-selectin and vascular cell adhesion protein 1, promoting immune cell adhesion [193]. Their interaction with platelets can result in coagulation abnormalities, driving thrombin generation and microthrombosis, characteristic of DIC observed in severe COVID-19. Additionally, viral damage to ECs exposes subendothelial collagen and releases von Willebrand factor, both of which activate platelets [196].

#### Platelet activation

Platelets can interact with viral components through their receptors, including TLRs, NLRs, Fc receptors, and complement receptors [197]. Upon activation, platelets can sequester and eliminate pathogens directly while releasing substances that inhibit viral replication, thereby aiding in infection control [198]. However, excessive activation may induce microthrombi formation in vital organs, leading to conditions such as ARDS and myocardial infarction. In IAV-infected mouse models, activated platelets recruit and interact with neutrophils, regulating NET release and exacerbating coagulopathy as well as lung pathology [199]. This increase in platelet activation is often associated with thrombocytopenia, which serves as a poor prognostic indicator. For example, DENV enhances platelet adhesion and degradation, leading to hemorrhagic outcomes [200], while DBV targets glycoprotein VI to further exacerbate platelet activation and contribute to thrombocytopenia [201]. Similarly, SARS-CoV-2 infection is also linked to thrombocytopenia and an elevated mortality rate [190].

#### Activation of the complement system

The complement system, bridging innate and adaptive immune responses, comprises over 30 types of soluble plasma proteins and membrane-associated proteins. Sensitive to cellular damage and pathogens, it can be exploited by viruses for tissue invasion. However, excessive complement activation during viral sepsis can trigger a “cytokine storm”, leading to endothelial dysfunction, thrombus formation, intravascular coagulation, and ultimately resulting in multiple organ failure and death. This is observed in severe cases of COVID-19 where high mortality rates correlate with extensive complement activation [202]. Similar detrimental effects of uncontrolled complement activity are noted in diseases such as influenza, MERS, and dengue [203–205].

#### Disruption of the coagulation system

Perturbations in the coagulation system are prevalent in acute infections such as Ebola, Lassa fever, yellow fever, hantavirus, DENV, and SARS-CoV-2. These disruptions, initially intended to limit viral spread, can induce thrombosis, secondary hemorrhage, and even DIC, leading to multi-organ dysfunction and increased mortality [153]. For instance, approximately 71% of COVID-19 fatalities met the DIC criteria [155]. IAV models also demonstrate the critical impact of coagulopathy on worsening pulmonary injury [146].

The pathophysiology of coagulation dysfunction in viral infections includes aberrant release of tissue factor and impairments in the protein C and S systems [206]. Viruses such as SARS-CoV, SARS-CoV-2, and EBOV induce expression of tissue factors in circulating blood cells, contributing to DIC and thrombosis [207]. HIV impairs the anticoagulant system by inducing protein S deficiency. Activated protein C, a plasma protease observed in severe sepsis, may exacerbate endothelial and epithelial cell dysfunction while influencing lung inflammation or survival during severe influenza [208]. Furthermore, activation of protease-activated receptor 1 by host thrombin enhances interactions between coagulation and inflammation, potentially increasing viral infectivity [209]. AMP-like peptides encoded by viruses, such as SARS-CoV-2, activate protease-activated receptor 2 to enhance coagulation [210]. These pathways culminate in widespread microvascular obstructions that lead to severe complications including AKI, ARDS, and stroke.

## Management and treatment strategies for viral sepsis

Management of viral sepsis integrates both etiological and supportive approaches. Unlike bacterial sepsis, for which antibiotics have been clinically proven effective, viral sepsis often lacks highly effective antiviral agents, leading to suboptimal outcomes. Care strategies primarily focus on symptomatic support to stabilize vital functions and manage secondary complications, frequently representing the only viable treatment option in clinical settings. Advanced therapeutic modalities such as immunomodulation, convalescent plasma (CP), and therapeutic plasma exchange (TPE), may potentially enhance patient outcomes by modulating the immune response.

### Supportive treatment

Supportive care can mitigate the risk of multi-organ failure and mortality in the management of sepsis, regardless of its etiology. The foundation of this care involves restoring microcirculation and addressing tissue hypoperfusion, particularly in severe cases of viral-induced sepsis [211]. Interventions include fluid resuscitation, vasopressor use, and blood transfusion. Patients with EVD, severe dengue, and DBV infections significantly benefit from prompt fluid resuscitation supplemented with vasopressors [19, 212]. In acute cases of EVD, blood transfusions are crucial for managing hemorrhagic complications.

As the disease progresses, supporting the respiratory, cardiac, and renal systems becomes paramount. Mechanical ventilation, including both non-invasive methods and early intubation, remains fundamental for respiratory support. In severe cases, extracorporeal membrane oxygenation has proven transformative, particularly for patients with refractory hypoxemia and severe myocardial depression [213, 214]. Extracorporeal membrane oxygenation has been associated with reduced mortality in conditions such as MERS, H1N1, and COVID-19 [215]. Additionally, renal replacement therapy assists patients with AKI by optimizing fluid balance [19, 216].

### Direct antiviral strategies

Most antiviral drugs are unsuitable for clinical use due to the lack of clinically proven efficacy against many prevalent viruses. Mechanistically, viruses, as obligate intracellular parasites, exploit host cellular mechanisms for their survival, complicating efforts to selectively target them adversely affecting host cells. The following sections provide a detailed overview of some antiviral drugs (Table 3) [217–236].

**Table 3:** Theoretical treatment strategies for viral sepsis

Targets of antiviral drugs	Name	Molecular mechanism	Target viruses	Stage of implementation	References
Inhibit viral attachment/Entry	Ceftazidime	Blocking spike protein–ACE2 interaction	SARS-CoV-2	Preclinical studies	[217, 218]
	hrsACE2 (APN01)	Binding the viral spike protein	SARS-CoV-2	In vitro cell experiments; Engineered organoids; Case report	[217, 218]
	Pyrimidine 2-12-2 and 3-110-22	Small molecule targeting SfS	Most flaviviruses (ZIKV and Japanese encephalitis virus)	Preclinical studies	[219]
	Pocapavir (V-073); Pleconaril; Vapendavir	Capsid-binding agents against enteroviruses; Inhibiting virion uncoating	Enterovirus	Enterovirus (FDA unapproved) Poliovirus (phase IIa)	[220, 221]
	Chloroquine (CQ); Hydroxychloroquine (HCQ)	Small molecule targeting endocytosis and other pathways	Flaviviruses; Retroviruses; Coronaviruses	Clinical drug for nonviral disease; FDA unapproved for COVID-19	[219]
Inhibit fusion	CA45, ADI-15878, and ADI-15742	Antibody targeting FP	EBOV	Preclinical studies	[222]
	HR2 analogues	Peptide targeting HR1	SARS-CoV-2; SARS-CoV; MERS-CoV	Preclinical studies	[223]
	EK1 and EK1C4	Peptide targeting HR1	SARS-CoV-2	Preclinical studies	[224]
	Arbidol (umifenovir IV)	Small molecule; Broad antiviral activity	IAV, RSV, adenovirus, EBOV, HBV, HCV, SARS-CoV-2	IAV (approved in Russia, China); SARS-CoV-2	[219]
Target host cell receptors	DAS181 (Fludase)	Small molecule targeting sialic acid (IAV receptor)	IAV	Phase II clinical studies	[225]
	Tamoxifen; Clomiphene; Raloxifene	Small molecule targeting; Estrogen receptor	SARS-CoV-2; HCV	FDA-approved SERMs; HCV (tamoxifen, phase I); SARS-CoV-2 (raloxifene, phase II)	[226]
Polymerase inhibitors (inhibit viral replication)	Remdesivir	Small molecule inhibiting the RdRp	EBOV, RSV, SARS-CoV, MERS-CoV, and SARS-CoV-2	Emergency use authorization for SARS-CoV-2	[219]
	Favipiravir	Small molecule (nucleoside analog) inhibiting the RdRp	LASV, EBOV, ZIKV, IAV, and DBV	DBV (phase III); Influenza [phase III (approved in Japan)]; EBOV (phase II); SARS-CoV-2 (phase III)	[227, 228]
	Zidovudine and lamivudine	Small molecule inhibiting the RdDp	HIV; HBV	FDA approved for HIV treatment	[229]
	Ribavirin	Small molecule inhibiting the RdDp and DdDp	RSV, IAVs, LASV	HCV, RSV (FDA approved) LASV (phase II) SARS-CoV-2 (phase II)	[219]
	VV116	Small molecule (remdesivir derivative) inhibiting the RdRp	SARS-CoV-2; RSV	SARS-CoV-2 (phase III)	[230]
	Paxlovid		HCV; SARS-CoV-2	FDA approved	[231]
	Molnupiravir (lagevrio)	Cytosine analogue	RSV; SARS-CoV-2	RSV (phase II); SARS-CoV-2 (FDA unapproved)	[228]
	Tembexa (brincidofovir)	Nucleoside analog	Smallpox virus; EBOV; CMV; Adenovirus	Smallpox virus (FDA approved); EBOV (phase II); CMV (phase III); Adenovirus (phase II)	[228]
Viral protein synthesis inhibitors	Fomivirsen (ISIS 2922)	Antisense oligonucleotide	CMV	Clinic use	[232]
Inhibitors of viral assembly (inhibit viral protease)	Disulfiram; Ebselen; Carmofur; Boceprevir	Small molecule inhibiting Protease	Coronaviruses	Disulfiram and Ebselen (phase II); Clinical drug for HCV	[219]
	Atazanavir	Small molecule inhibiting Protease	Retroviruses, HIV	FDA approved for HIV	[219]
	Tocilizumab and anakinra	Targeting interleukin-6 receptor to treat cytokine release syndrome	SARS-CoV-2	FDA-approved for hospitalized COVID-19 patients	[233, 234]
	Ritonavir; Lopinavir	Small molecule targeting the Protease	HIV; SARS-CoV-1; SARS-CoV-2	HIV (FDA approved); SARS-CoV-1, SARS-CoV-2 (completed phase II/III, not recommended)	[235, 236]
Inhibitors of viral release	Oseltamivir; Zanamivir	Small molecule blocking the viral membrane protein	IVA	WHO-approved	[219]

*ACE2* angiotensin-converting enzyme 2, *Phase I or II* Phase I or II clinical trial, *HIV* human immunodeficiency virus, *SARS-CoV-2* severe acute respiratory syndrome coronavirus 2, *IAV* influenza A virus, *HCV* hepatitis C virus, *EBOV* Ebola virus, *ZIKV* Zika virus, *MERS-CoV* Middle East respiratory syndrome coronavirus, *DENV* Dengue virus, *LASV* Lassa virus, *RSV* respiratory syncytial adenovirus, *HBV* hepatitis B virus, *HR1* heptad repeat 1, *SfS* surface subunit, *FP* fusion peptide, *FDA* U.S. Food and Drug Administration, *WHO* World Health Organization, *DBV* Dabie bandavirus, *COVID-19* coronavirus disease 2019

#### The inhibitors of virus attachment/entry

This class of antiviral agents targets host receptors, co-receptors, or viral spike proteins to inhibit viral attachment and entry, thereby disrupting the early stages of the viral replication cycle.

DAS181 (Fludase) is a sialidase fusion protein that enzymatically cleaves sialic acids from the host respiratory epithelium, thereby blocking viral entry. Preclinical and clinical studies have shown that DAS181 possesses broad-spectrum antiviral activity without inducing cellular toxicity against various strains of influenza, including H1N1 and avian influenza [225, 237]. It effectively inhibits the replication of H7N9 and its oseltamivir-resistant R292K variants in mice, achieving a 100% survival rate in lethally infected mice when administered daily shortly after infection onset. In a phase III clinical trial involving hospitalized patients with parainfluenza virus infections, DAS181 significantly reduced viral load and improved pulmonary function [237]. These findings position DAS181 as a promising candidate for therapeutic intervention.

Azedoxifene acetate, a selective estrogen receptor modulator, demonstrates potential efficacy against coronaviruses. It functions as an entry inhibitor by targeting the post-attachment penetration stage. This mechanism effectively inhibits pseudovirus infections of SARS-CoV-2, including the Delta and Omicron variants, as well as SARS-CoV, while also reducing IL-6 levels both in vitro and in vivo [226].

Fostemsavir (brand name Rukobia), a recently Food and Drug Administration (FDA)-approved attachment inhibitor, is a prodrug of temsavir. Upon metabolism, it binds to the gp120 subunit of the HIV-1 envelope glycoprotein gp160, thereby effectively inhibiting viral attachment to CD4 receptors in the host immune system [238]. In the phase III trial (NCT02362503), patients with advanced HIV-1 disease and limited therapeutic options who received fostemsavir exhibited a significantly greater decrease in HIV-1 RNA levels compared to those receiving placebo treatment. This efficacy was maintained over 48 weeks. Additionally, fostemsavir was generally well tolerated and demonstrated increasing virological and immunological response rates up to 96 weeks [239].

Human recombinant soluble angiotensin-converting enzyme 2 (ACE2), targeting the ACE2 receptor to block entry of SARS-CoV-2 Spike (S) protein, exhibits therapeutic potential for reducing viral loads and protecting organoids. This effectiveness has been observed during treatment of a female COVID-19 patient, and confirmed through phase I and II trials involving both healthy volunteers and COVID-19 patients [217, 218].

#### Fusion inhibitors

Membrane fusion inhibitors, which target enveloped viruses, act on components such as the fusion peptide and heptad repeat (HR) 1 and 2 to obstruct viral entry. Antibodies like CA45, ADI-15878, and ADI-15742 [222], along with hydrophilically enhanced HR2 analogs, have demonstrated efficacy against viruses including EBOV and MERS-CoV, significantly reducing viral titers in mice by over 1000-fold [223, 240]. Pan-coronavirus inhibitors that target HR1 domains have shown substantial inhibition of SARS-CoV-2 membrane fusion in mouse models [224]. Specific pyrimidine compounds targeting flavivirus E proteins, such as pyrimidine 2-12-2 and 3-110-22, inhibit membrane fusion and have exhibited the ability to counteract infections like ZIKV and Japanese encephalitis virus in vitro [219].

Chloroquine and hydroxychloroquine (HCQ), while influencing endocytosis and various stages of viral assembly and replication in flaviviruses, retroviruses, and coronaviruses [219], demonstrate a lack of clinical efficacy against coronaviruses. Pre-exposure to HCQ among healthcare workers did not reduce the risk of SARS-CoV-2 infection and was associated with an increased incidence of adverse events [241, 242]. Larger trials have indicated that HCQ use is linked to higher mortality rates [243].

#### Viral replication inhibitors

RNA-dependent RNA polymerase (RdRp) and DNA polymerase are pivotal in the replication of genomic material (DNA or RNA) and serve as prime targets for antiviral strategies. Remdesivir, an adenosine analog that targets RdRp, has demonstrated therapeutic potential against a broad spectrum of viruses, including EBOV, RSV, SARS-CoV, MERS-CoV, and SARS-CoV-2. Research indicate that remdesivir effectively mitigates the progression of ARDS and reduces mortality in SARS-CoV-2 infections [244, 245]. A 3-day course of remdesivir has been shown to reduce the risk of hospitalization or death in non-hospitalized patients [246]. Favipiravir, another RdRp inhibitor, has yielded promising results in clinical trials but is not yet widely approved (Table 3) [227, 228]. Clinical study suggests that favipiravir can facilitate rapid viral clearance in influenza cases and significantly lower mortality rates among DBV-infected patients [227].

VV116, an oral nucleoside antiviral approved in Uzbekistan for targeting the RdRp of SARS-CoV-2, shows significant antiviral activity against various strains of SARS-CoV-2, including the Omicron variant, while exhibiting no genotoxicity in preclinical evaluations. A phase III trial (NCT05341609) conducted in China revealed that VV116 has comparable efficacy to PAXLOVID (nirmatrelvir packaged with ritonavir), with individuals treated with VV116 experiencing faster clinical recovery and fewer safety concerns. Other FDA-approved RdRp inhibitors include molnupiravir and azvudine, underlining the therapeutic potential of this target mechanism in adult patients with mild-to-moderate COVID-19 [247].

#### Viral protease inhibitors

Viral replication necessitates post-translational modifications mediated by proteases. In coronaviruses, the main protease (M^pro^ or 3CL^pro^) plays a crucial role. Identified inhibitors of CoV M^pro^ include disulfiram, ebselen, carmofur, and notably boceprevir, which were originally approved for HCV [168]. Other hepatitis C protease inhibitors, such as telaprevir and simeprevir, have also undergone clinical validation.

Nirmatrelvir, an oral M^pro^ inhibitor, impedes viral replication while ritonavir enhances its plasma stability by inhibiting metabolism through the cytochrome P-450 3A4 (CYP3A4) pathway. The phase II/III evaluation of protease inhibition for COVID-19 in high-risk patients (EPIC-HR) trial demonstrated that nirmatrelvir/ritonavir reduces the risk of mortality and accelerates viral load reduction in high-risk outpatient COVID-19 patients, with greater benefits observed in individuals exhibiting more severe risk factors. Further studies are needed to identify the populations most likely to benefit [248].

Additionally, SY110 has shown efficacy in reducing viral loads across various coronaviruses in animal models. Olgotrelvir (STI-1558), a dual inhibitor of SARS-CoV-2 M^pro^ and cathepsin L, exhibited a favorable safety profile and antiviral activity in phase I trials, emerging as a potent oral antiviral candidate [249]. Ensitrelvir (S-217622), targeting SARS-CoV-2’s main protease, significantly lowers viral load and mitigates disease severity in hamsters infected with SARS-CoV-2 [250].

### Immunomodulatory therapy

Sepsis is characterized by an imbalance in the immune system, manifesting as excessive inflammation and immunosuppression. Therapeutic strategies are designed to rectify this imbalance, constituting a crucial component of supportive treatment for viral sepsis.

#### Cytokine antagonists and interferon therapy

Cytokines such as IL-1, IL-6, TNF, and IFNs serve as key inflammatory mediators in viral infections. Anti-cytokine therapies, including IL-1 inhibitors (like anakinra) and IL-6 inhibitors (such as tocilizumab and sarilumab), effectively mitigate inflammation in severe cases of COVID-19. Anakinra has been shown to reduce venous and arterial complications in critically ill patients [251], while tocilizumab significantly decreases mortality and intubation rates among ARDS patients [252]. Additionally, TNF neutralization has been found to reduce neurotoxicity and mortality in DENV-infected mice [253]. However, both anakinra and tocilizumab do not significantly shorten clinical recovery times [254]. Furthermore, anti-IL-6 therapies may impair viral clearance and increase susceptibility to secondary infections, necessitating rigorous monitoring for respiratory and infectious complications [255, 256].

Type I IFNs, including IFN-α and IFN-β, are essential for antiviral defense; however, insufficient levels can exacerbate infections [257]. Early administration of IFNs has shown protective effects against lethal SARS-CoV and MERS-CoV infections in animal models [231, 233]. A retrospective cohort study found that early administration of IFN-α2b (within 5 d of admission) is associated with reduced in-hospital mortality among COVID-19 patients, whereas delayed administration correlates with increased mortality and offers no significant benefit to moderately ill patients [258]. Furthermore, excessive levels of IFN may trigger autoimmune or inflammatory conditions [257]. Therefore, careful monitoring of IFN therapy is essential to manage potential adverse effects.

#### Glucocorticoids

Corticosteroids are typically used to modulate host cell-mediated immune responses and inflammatory cytokines in various infectious diseases [259]. However, their use should be restricted to specific situations for certain viral infections, such as SARS-CoV-2 with hypoxemia, rather than being applied broadly outside these indications.

Dexamethasone, a preferred glucocorticoid, significantly reduces 28-day mortality in severe COVID-19 patients requiring respiratory support [260, 261]. Its administration is critical during the progression to ARDS in COVID-19 patients [262] and is also utilized in the treatment of SARS, influenza, varicella zoster, and HSV encephalitis [42, 263]. In critically ill COVID-19 patients, corticosteroids can decrease both mortality and hospitalization days without increasing the risk of secondary infections, as observed in cases of SARS and influenza.

Nevertheless, the long-term consequences of dexamethasone, including hypertension, cardiac hypertrophy, and hyperglycemia, necessitate careful monitoring of blood pressure and hemoglobin A1c levels throughout treatment [264]. Additionally, its immunosuppressive properties warrant caution in conditions such as dengue fever and influenza [263].

#### Immune enhancers

In response to the detrimental effects of immunosuppression in viral sepsis, immunostimulants such as GM-CSF and IL-7 are garnering increasing attention. GM-CSF has potential applications in alleviating pulmonary complications [265], while IL-7 enhances T cell responses to both HIV-1 and SARS-CoV-2 [266]. The programmed death-1 pathway, which is implicated in sepsis-induced T cell dysfunction and mortality [42], is also emerging as a promising therapeutic target, particularly in severe influenza cases [267]. Overall, the therapeutic strategy for viral sepsis is evolving towards a synergistic approach that integrates anti-inflammatory effects with immune enhancement.

#### Cell therapy

Cellular therapies, including T cell-, NK cell-, and mesenchymal stem cell-based therapies, offer promising treatment options for viral infections. These approaches utilize engineered immune cells to target infected cells and enhance antiviral responses without exacerbating systemic inflammation. NK cells naturally detect and neutralize infected cells without prior exposure, while virus-specific T cells bolster targeted antiviral defenses with minimal inflammatory response [268]. Mesenchymal stem cell-based therapies exhibit a range of beneficial properties, including antimicrobial, anti-inflammatory, regenerative, angiogenic, antifibrotic, antioxidative, and anti-apoptotic effects, and have been extensively studied in clinical settings. A systematic review assessing the therapeutic potential and safety of various stem cell types for COVID-19 and ARDS revealed encouraging results [269]. Although these studies did not specifically address MERS or SARS, they consistently highlighted the advantages of stem cell therapy in modulating immune and inflammatory pathways associated with lung damage in patients suffering from COVID-19 and ARDS [270].

### CP treatment

CP therapy, which involves transfusing plasma from recovered patients into infected individuals, can provide specific antibodies that neutralize viral agents and alleviate septic symptoms. It has demonstrated efficacy during various viral outbreaks, including SARS, MERS, Ebola, and certain strains of influenza [271, 272]. For instance, during the SARS epidemic, meta-analyses suggested that CP therapy significantly reduced both viral loads and mortality rates [273]. Additionally, it has proven effective in reducing mortality rates associated with Ebola [274] and has shown benefits in H7N9 infections where conventional treatments have failed [275]. Plasma enriched with H5N1 antibodies has also been found to decrease the risk of mortality [276].

Despite historical successes, the application of CP therapy in COVID-19 requires careful interpretation [275]. Observational studies have indicated that CP is generally safe for COVID-19 patients and may reduce viral loads as well as coagulation disorders [276–278]. However, an exploratory analysis involving 4330 patients revealed no significant benefits in reducing mortality at 7 d. Furthermore, a large-scale randomized controlled trial revealed that CP therapy did not significantly impact the progression to severe COVID-19, clinical status, or overall mortality [278, 279]. These findings highlight the variability in CP’s effectiveness and underscore the necessity for further research to elucidate its role in treating viral infections.

### TPE

TPE functions by excreting toxic compounds and small viral constituents, while enhancing endothelial activity. It surpasses conventional methods such as heparin therapy and significantly reduces the incidence of ARDS, AKI, bleeding episodes, and other sepsis-associated complications [280]. In 2009, TPE was demonstrated to be a life-saving intervention for pediatric patients suffering from H1N1-induced acute lung injury and shock [281]. Additionally, lectin affinity plasmapheresis has achieved up to 80% clearance of MERS-CoV within a 3-hour window in vitro [282]. A meta-analysis further highlighted TPE’s potential in rectifying coagulative disorders and diminishing mortality among COVID-19 patients, particularly when combined with antithrombin and tissue fibrinogen inhibitors derived from fresh frozen plasma [283].

### Prevention of secondary infections

Secondary infections, including bacterial, fungal, or viral pathogens, complicate primary viral ailments, particularly in patients with viral sepsis who experience immune suppression. These secondary infections can result in severe outcomes such as respiratory failure, shock, extended ICU stays, and increased mortality [284]. During the influenza pandemic, secondary bacterial infections significantly contributed to elevated mortality rates [285]. Similarly, MERS-CoV and SARS-CoV-2 infections have been aggravated by secondary infections, leading to prolonged hospitalizations, multi-organ failure, and heightened mortality rates [286]. Additionally, cases of SFTS complicated by secondary Aspergillus infections exhibit a high 28-day mortality rate [287].

Common pathogens associated with influenza include *Streptococcus pneumoniae*, *Staphylococcus aureus*, and *Haemophilus influenzae*. In COVID-19, respiratory infections often involve *Staphylococcus aureus*, *Haemophilus influenzae*, *Acinetobacter*, *Klebsiella*, and *Pseudomonas* species, while bloodstream infections may involve *Escherichia coli* and *Staphylococcus aureus.* Other significant secondary pathogens in severe cases encompass *Mycoplasma pneumoniae*, *Chlamydia pneumoniae*, and *Legionella pneumophila* [288].

However, the prolonged use of antibiotics to target these pathogens has contributed to increased antimicrobial resistance [289], complicating treatment strategies and elevating patient risk. Effective management of secondary infections requires an integrated approach that emphasizes judicious and timely use of antimicrobials alongside rapid diagnostic techniques. Collecting high-quality microbiological samples, such as bronchoalveolar lavage fluid, is crucial for accurate pathogen identification and tailored treatment. A strategic evidence-based approach is essential for combating secondary infections in severe viral cases while reducing overall mortality.

## Evolving landscape of viral sepsis: challenges and future directions

Identifying viral sepsis presents significant challenges due to the potential for bacterial co-infection and the inconsistent application of the term “viral sepsis”, complicating both prospective and retrospective diagnostics. Prospective diagnostic efforts are often hindered by uncertainties regarding bacterial co-infections in critically ill patients, while retrospective methods, which frequently rely on billing codes, may fail to accurately capture the incidence or characteristics of viral sepsis. These challenges highlight the urgent need for standardized criteria and tools specifically designed to identify viral sepsis.

The similar clinical presentations and overlapping inflammatory responses between viral and bacterial infections further complicate diagnosis, particularly when bacterial infections occur after viral ones. Although advanced diagnostic tools for viral diseases exist, they are often constrained by issues related to sensitivity, specificity, and availability in resource-limited settings. Additionally, the high costs associated with these diagnostics and the mutable nature of RNA viruses introduce further complexity. The emergence of viruses such as SARS-CoV-2 emphasizes the necessity for adaptable diagnostic techniques that can keep pace with viral evolution. Therefore, developing standardized diagnostic criteria and screening tools for viral sepsis would enhance diagnostic precision, improve patient outcomes, and strengthen epidemiological data.

Challenges also persist in the management and treatment of viral sepsis. Despite significant advancements in understanding the signaling pathways and molecular mechanisms involved, this field remains contentious. Viruses interact with a wide range of host signaling pathways, often in ways that differ among various pathogens, making it difficult to generalize findings from one viral infection to another. Moreover, viruses are adept at manipulating host pathways, complicating efforts to identify therapeutic targets without disrupting normal cellular functions. These challenges are further exacerbated by variable clinical outcomes and organ responses.

The future of managing viral sepsis presents several opportunities for improvement. Regardless of whether sepsis is induced by bacteria, viruses, parasites, or fungi, timely detection and intervention can prevent progression to septic shock and multi-organ failure. This review explores the epidemiology, clinical manifestations, and complex signaling pathways associated with organ damage in sepsis, along with molecular dynamics and diagnostic challenges. By deepening the understanding of viral interactions, these insights will enhance both public awareness and professional knowledge, while improving prevention and treatment strategies.

The complexity of viral sepsis has also catalyzed advancements in medical treatments, including immunomodulatory therapies such as CP and innovations in nanomedicine. These approaches aim to modulate inflammatory responses and provide broad-spectrum antiviral effects, holding promise for future therapeutic developments. Although many of these treatments remain in the early stages of research, the integrated strategy of combining organ support with immunomodulation shows potential benefits for critically ill patients.

In summary, ongoing efforts to elucidate the complexities of viral pathogens, refine therapeutic strategies, and translate preclinical findings into clinical practice are poised to revolutionize the management of viral sepsis. The future appears promising, with advanced antivirals and multifaceted therapies converging to offer comprehensive and effective treatment options.

## Data Availability

The datasets used and/or analyzed during the current study are available from the corresponding author on reasonable request.

## Abbreviations

ACE2: Angiotensin-converting enzyme 2
AIM2: Absent in melanoma 2
AKI: Acute kidney injury
ARDS: Acute respiratory distress syndrome
ASC: Apoptosis-associated speck-like protein containing a caspase-recruitment domain
ATF4: Activating transcription factor 4
BBB: Blood–brain barrier
cGAS: GMP-AMP synthase
CHOP: C/EBP homologous protein
CMV: Cytomegalovirus
CNS: Central nervous system
COVID-19: Coronavirus disease 2019
CP: Convalescent plasma
DBV: Dabie bandavirus
DENV: Dengue virus
DIC: Disseminated intravascular coagulation
EBOV: Ebola virus
EBV: Epstein-Barr virus
ECs: Endothelial cells
ER: Endoplasmic reticulum
EVD: Ebola virus disease
FADD: FAS-associated protein with death domain
GPX4: Glutathione peroxidase 4
GM-CSF: Granulocyte–macrophage colony-stimulating factor
GMP-AMP: Guanosine monophosphate adenosine monophosphate
HBV: Hepatitis B virus
HCV: Hepatitis C virus
HCQ: Hydroxychloroquine
HFRS: Hemorrhagic fever with renal syndrome
HIV: Human immunodeficiency virus
HPS: Hantavirus pulmonary syndrome
HSV: Herpes simplex virus
IAV: Influenza A virus
ICU: Intensive Care Unit
IL: Interleukin
IRF: Interferon regulatory factor
LGP2: Laboratory of genetics and physiology 2
MAVS: Mitochondrial antiviral signaling
MDA5: Melanoma differentiation-associated protein 5
MDSC: Myeloid-derived suppressor cell
MERS: Middle East respiratory syndrome
mtDAMPs: Mitochondrial damage-associated molecular patterns
NK: Natural killer
NLRs: Nucleotide-binding and oligomerization domain (NOD)-like receptors
NS1: Nonstructural protein 1
ORF: Open reading frame
OXPHO: Oxidative phosphorylation
PCR: Polymerase chain reaction
PRRs: Pattern recognition receptors; retinoic acid-inducible gene-I
RIG-I: Retinoic acid-inducible gene-I
RIPK3: Receptor-interacting protein kinase 3
RdRp: RNA-dependent RNA polymerase
RLRs: RIG-I-like receptors
ROS: Reactive oxygen species
RSV: Respiratory syncytial virus
SARS-CoV-2: Severe acute respiratory syndrome coronavirus 2
SFTS: Severe fever with thrombocytopenia syndrome
SOFA: Sequential Organ Failure Assessment
STING: Stimulator of interferon genes
TBK1: TRAF family member-associated NF-κB activator (TANK)-binding kinase 1
TIR: Toll/interleukin-1 receptor/resistance protein
TLRs: Toll-like receptors
TPE: Therapeutic plasma exchange
TNF: Tumor necrosis factor
WNV: West Nile virus
ZBP1: Z-DNA binding protein 1

## References

[1] Singer M, Deutschman CS, Seymour CW, Shankar-Hari M, Annane D, Bauer M, et al. The third international consensus definitions for sepsis and septic shock (sepsis-3). JAMA. 2016;315(8):801–10. 26903338 10.1001/jama.2016.0287 PMC4968574

[2] Gong T, Liu YT, Fan J. Exosomal mediators in sepsis and inflammatory organ injury: unraveling the role of exosomes in intercellular crosstalk and organ dysfunction. Mil Med Res. 2024;11(1):24. 38644472 10.1186/s40779-024-00527-6 PMC11034107

[3] Lelubre C, Vincent JL. Mechanisms and treatment of organ failure in sepsis. Nat Rev Nephrol. 2018;14(7):417–27. 29691495 10.1038/s41581-018-0005-7

[4] Rhee C, Dantes R, Epstein L, Murphy DJ, Seymour CW, Iwashyna TJ, et al. Incidence and trends of sepsis in us hospitals using clinical vs. claims data, (2009–2014). JAMA. 2017;318(13):1241–9. 28903154 10.1001/jama.2017.13836 PMC5710396

[5] Yuki K, Koutsogiannaki S. Pattern recognition receptors as therapeutic targets for bacterial, viral and fungal sepsis. Int Immunopharmacol. 2021;98:107909. 34182242 10.1016/j.intimp.2021.107909 PMC8380728

[6] GBD 2019 Antimicrobial Resistance Collaborators. Global mortality associated with 33 bacterial pathogens in 2019: a systematic analysis for the global burden of disease study 2019. Lancet. 2022;400(10369):2221–48. 36423648 10.1016/S0140-6736(22)02185-7 PMC9763654

[7] Yang H, Rao Z. Structural biology of SARS-COV-2 and implications for therapeutic development. Nat Rev Microbiol. 2021;19(11):685–700. 34535791 10.1038/s41579-021-00630-8 PMC8447893

[8] Pinto AK, Ramos HJ, Wu X, Aggarwal S, Shrestha B, Gorman M, et al. Deficient IFN signaling by myeloid cells leads to MAVS-dependent virus-induced sepsis. PLoS Pathog. 2014;10(4):e1004086. 24743949 10.1371/journal.ppat.1004086 PMC3990718

[9] Evans T. Diagnosis and management of sepsis. Clin Med (Lond). 2018;18(2):146–9. 29626019 10.7861/clinmedicine.18-2-146 PMC6303466

[10] Shappell CN, Klompas M, Chan C, Chen T, Kanjilal S, McKenna C, et al. Use of electronic clinical data to track incidence and mortality for SARS-CoV-2-associated sepsis. JAMA Netw Open. 2023;6(9):e2335728. 37773495 10.1001/jamanetworkopen.2023.35728 PMC10543118

[11] Shappell C, Rhee C, Klompas M. Update on sepsis epidemiology in the era of COVID-19. Semin Respir Crit Care Med. 2023;44(1):173–84. 36646093 10.1055/s-0042-1759880

[12] Huson MA, Grobusch MP, van der Poll T. The effect of hiv infection on the host response to bacterial sepsis. Lancet Infect Dis. 2015;15(1):95–108. 25459220 10.1016/S1473-3099(14)70917-X

[13] Peng JM, Du B, Qin HY, Wang Q, Shi Y. Metagenomic next-generation sequencing for the diagnosis of suspected pneumonia in immunocompromised patients. J Infect. 2021;82(4):22–7. 33609588 10.1016/j.jinf.2021.01.029

[14] Fozouni P, Son S, de León D, Derby M, Knott GJ, Gray CN, Dambrosio MV, et al. Amplification-free detection of SARS-CoV-2 with CRISPR-CAS13a and mobile phone microscopy. Cell. 2021;184(2):323-33.e9. 33306959 10.1016/j.cell.2020.12.001 PMC7834310

[15] Rauch JN, Valois E, Ponce-Rojas JC, Aralis Z, Lach RS, Zappa F, et al. Comparison of severe acute respiratory syndrome coronavirus 2 screening using reverse transcriptase-quantitative polymerase chain reaction or CRISPR-based assays in asymptomatic college students. JAMA Netw Open. 2021;4(2):e2037129. 33570576 10.1001/jamanetworkopen.2020.37129 PMC7879237

[16] Kaminski MM, Abudayyeh OO, Gootenberg JS, Zhang F, Collins JJ. CRISPR-based diagnostics. Nat Biomed Eng. 2021;5(7):643–56. 34272525 10.1038/s41551-021-00760-7

[17] Michelin L, Bellei N, da Costa F, Gomes M, Raboni SM, Kairalla M, Correa RA, et al. Respiratory syncytial virus: challenges in diagnosis and impact on the elderly: Recommendations from a multidisciplinary panel. Hum Vaccin Immunother. 2024;20(1):2388943. 39161095 10.1080/21645515.2024.2388943 PMC11340750

[18] Carrico J, Hicks KA, Wilson E, Panozzo CA, Ghaswalla P. The annual economic burden of respiratory syncytial virus in adults in the United States. J Infect Dis. 2024;230(2):e342–52. 38060972 10.1093/infdis/jiad559 PMC11326840

[19] Minozzi S, Lytras T, Gianola S, Gonzalez-Lorenzo M, Castellini G, Galli C, et al. Comparative efficacy and safety of vaccines to prevent seasonal influenza: a systematic review and network meta-analysis. EClinicalMedicine. 2022;46:101331. 35360146 10.1016/j.eclinm.2022.101331 PMC8961170

[20] The burden of influenza. 30 Mar 2024. Available at: https://www.who.int/news-room/feature-stories/detail/the-burden-of-influenza. Sep 2024

[21] Middle East respiratory syndrome coronavirus - kingdom of Saudi Arabia. 16 Feb 2024. Available at: https://www.who.int/emergencies/disease-outbreak-news/item/2024-DON506. Sep 2024

[22] Middle East respiratory syndrome: global summary and assessment of risk - 16 Nov 2022. 16 Nov 2022. Available at: https://www.who.int/publications/i/item/WHO-MERS-RA-2022. Sep 2024

[23] Number of COVID-19 cases reported to WHO. 17 Oct 2024. Available at: https://data.who.int/dashboards/COVID19/cases?n=c. Oct 2024

[24] Dengue and severe dengue. 23 Apr 2024. Available at: https://www.who.int/news-room/fact-sheets/detail/dengue-and-severe-dengue. Sep 2024

[25] Zeng Z, Zhan J, Chen L, Chen H, Cheng S. Global, regional, and national dengue burden from 1990 to 2017: a systematic analysis based on the global burden of disease study 2017. EClinicalMedicine. 2021;32:100712. 33681736 10.1016/j.eclinm.2020.100712 PMC7910667

[26] Dengue - global situation. 30 May 2024. Available at: https://www.who.int/emergencies/disease-outbreak-news/item/2024-DON518. Sep 2024

[27] Cui H, Shen S, Chen L, Fan Z, Wen Q, Xing Y, et al. Global epidemiology of severe fever with thrombocytopenia syndrome virus in human and animals: a systematic review and meta-analysis. Lancet Reg Health West Pac. 2024;48:101133. 39040038 10.1016/j.lanwpc.2024.101133 PMC11261768

[28] Park SW, Lee CS, Kim JH, Bae IG, Moon C, Kwak YG, et al. Severe fever with thrombocytopenia syndrome: comparison with scrub typhus and clinical diagnostic prediction. BMC Infect Dis. 2019;19(1):174. 30782137 10.1186/s12879-019-3773-1 PMC6381645

[29] Zhang H, Zhang L. Knowledge mapping of severe fever with thrombocytopenia syndrome: a bibliometric analysis. Front Microbiol. 2024;15:1423181. 39139373 10.3389/fmicb.2024.1423181 PMC11319145

[30] Jacob ST, Crozier I, Fischer WA 2nd, Hewlett A, Kraft CS, Vega MA, et al. Ebola virus disease. Nat Rev Dis Primers. 2020;6(1):13. 32080199 10.1038/s41572-020-0147-3 PMC7223853

[31] Ebola virus disease. 20 Apr 2023. Available at: https://www.who.int/news-room/fact-sheets/detail/ebola-virus-disease. Sep 2024.

[32] Prescott JB, Marzi A, Safronetz D, Robertson SJ, Feldmann H, Best SM. Immunobiology of Ebola and Lassa virus infections. Nat Rev Immunol. 2017;17(3):195–207. 28111475 10.1038/nri.2016.138

[33] Basinski AJ, Fichet-Calvet E, Sjodin AR, Varrelman TJ, Remien CH, Layman NC, et al. Bridging the gap: using reservoir ecology and human serosurveys to estimate lassa virus spillover in West Africa. PLoS Comput Biol. 2021;17(3):e1008811. 33657095 10.1371/journal.pcbi.1008811 PMC7959400

[34] Brocato RL, Hooper JW. Progress on the prevention and treatment of hantavirus disease. Viruses. 2019;11(7):610. 31277410 10.3390/v11070610 PMC6669544

[35] Avšič-Županc T, Saksida A, Korva M. Hantavirus infections. Clin Microbiol Infect. 2019;21:e6–16. 10.1111/1469-0691.12291 24750436

[36] Vial PA, Ferrés M, Vial C, Klingström J, Ahlm C, López R, et al. Hantavirus in humans: a review of clinical aspects and management. Lancet Infect Dis. 2023;23(9):e371–82. 37105214 10.1016/S1473-3099(23)00128-7

[37] Lassa fever. 31 Jul 2017. Available at: https://www.who.int/news-room/fact-sheets/detail/lassa-fever. Sep 2024.

[38] Raabe V, Mehta AK, Evans JD. Lassa virus infection: a summary for clinicians. Int J Infect Dis. 2022;119:187–200. 35395384 10.1016/j.ijid.2022.04.004

[39] Gu X, Zhou F, Wang Y, Fan G, Cao B. Respiratory viral sepsis: epidemiology, pathophysiology, diagnosis and treatment. Eur Respir Rev. 2020;29(157):200038. 32699026 10.1183/16000617.0038-2020 PMC9489194

[40] Cillóniz C, Dominedò C, Magdaleno D, Ferrer M, Gabarrús A, Torres A. Pure viral sepsis secondary to community-acquired pneumonia in adults: risk and prognostic factors. J Infect Dis. 2019;220(7):1166–71. 31115456 10.1093/infdis/jiz257 PMC7107497

[41] Nguyen-Van-Tam JS, O’Leary M, Martin ET, Heijnen E, Callendret B, Fleischhackl R, et al. Burden of respiratory syncytial virus infection in older and high-risk adults: a systematic review and meta-analysis of the evidence from developed countries. Eur Respir Rev. 2022;31(166):220105. 36384703 10.1183/16000617.0105-2022 PMC9724807

[42] Lin GL, McGinley JP, Drysdale SB, Pollard AJ. Epidemiology and immune pathogenesis of viral sepsis. Front Immunol. 2018;9:2147. 30319615 10.3389/fimmu.2018.02147 PMC6170629

[43] Kalil AC, Thomas PG. Influenza virus-related critical illness: pathophysiology and epidemiology. Crit Care. 2019;23(1):258. 31324202 10.1186/s13054-019-2539-x PMC6642581

[44] Tokars JI, Olsen SJ, Reed C. Seasonal incidence of symptomatic influenza in the united states. Clin Infect Dis. 2018;66(10):1511–8. 29206909 10.1093/cid/cix1060 PMC5934309

[45] Yang Y, Guo F, Zhao W, Gu Q, Huang M, Cao Q, et al. Novel avian-origin influenza A (H7N9) in critically ill patients in China*. Crit Care Med. 2015;43(2):339–45. 25365721 10.1097/CCM.0000000000000695

[46] Reed C, Chaves SS, Perez A, D’Mello T, Daily Kirley P, Aragon D, et al. Complications among adults hospitalized with influenza: a comparison of seasonal influenza and the 2009 H1N1 pandemic. Clin Infect Dis. 2014;59(2):166–74. 24785230 10.1093/cid/ciu285 PMC7314251

[47] McChlery S, Ramage G, Bagg J. Respiratory tract infections and pneumonia. Periodontol 2000. 2009;49(1):151–65. 19152532 10.1111/j.1600-0757.2008.00278.x PMC7168030

[48] Chen B, Tian EK, He B, Tian L, Han R, Wang S, et al. Overview of lethal human coronaviruses. Signal Transduct Target Ther. 2020;5(1):89. 32533062 10.1038/s41392-020-0190-2 PMC7289715

[49] Lam CW, Chan MH, Wong CK. Severe acute respiratory syndrome: clinical and laboratory manifestations. Clin Biochem Rev. 2004;25(2):121–32. 18458712 PMC1904416

[50] Karakike E, Giamarellos-Bourboulis EJ, Kyprianou M, Fleischmann-Struzek C, Pletz MW, Netea MG, et al. Coronavirus disease 2019 as cause of viral sepsis: a systematic review and meta-analysis. Crit Care Med. 2021;49(12):2042–57. 34259663 10.1097/CCM.0000000000005195 PMC8594513

[51] Herminghaus A, Osuchowski MF. How sepsis parallels and differs from COVID-19. EBioMedicine. 2022;86:104355. 36470836 10.1016/j.ebiom.2022.104355 PMC9718536

[52] Zhou F, Yu T, Du R, Fan G, Liu Y, Liu Z, et al. Clinical course and risk factors for mortality of adult inpatients with COVID-19 in Wuhan, China: a retrospective cohort study. Lancet. 2020;395(10229):1054–62. 32171076 10.1016/S0140-6736(20)30566-3 PMC7270627

[53] Aguilar-Briseño JA, Moser J, Rodenhuis-Zybert IA. Understanding immunopathology of severe dengue: lessons learnt from sepsis. Curr Opin Virol. 2020;43:41–9. 32896675 10.1016/j.coviro.2020.07.010

[54] Teparrukkul P, Hantrakun V, Day NPJ, West TE, Limmathurotsakul D. Management and outcomes of severe dengue patients presenting with sepsis in a tropical country. PLoS ONE. 2017;12(4):e0176233. 28437459 10.1371/journal.pone.0176233 PMC5402971

[55] Maslow JN, Kwon JJ, Mikota SK, Spruill S, Cho Y, Jeong M. Severe fever and thrombocytopenia syndrome virus infection: considerations for vaccine evaluation of a rare disease. Hum Vaccin Immunother. 2019;15(10):2249–57. 31215838 10.1080/21645515.2019.1633875 PMC6816409

[56] de Greslan T, Billhot M, Rousseau C, Mac Nab C, Karkowski L, Cournac JM, et al. Ebola virus-related encephalitis. Clin Infect Dis. 2016;63(8):1076–8. 27418576 10.1093/cid/ciw469

[57] Southeast Asia Infectious Disease Clinical Research Network. Causes and outcomes of sepsis in Southeast Asia: a multinational multicentre cross-sectional study. Lancet Glob Health. 2017;5(2):e157–67. 28104185 10.1016/S2214-109X(17)30007-4 PMC5332551

[58] D’Souza MH, Patel TR. Biodefense implications of new-world hantaviruses. Front Bioeng Biotechnol. 2020;8:925. 32850756 10.3389/fbioe.2020.00925 PMC7426369

[59] Baillet N, Reynard S, Perthame E, Hortion J, Journeaux A, Mateo M, et al. Systemic viral spreading and defective host responses are associated with fatal Lassa fever in macaques. Commun Biol. 2021;4(1):27. 33398113 10.1038/s42003-020-01543-7 PMC7782745

[60] Okokhere P, Colubri A, Azubike C, Iruolagbe C, Osazuwa O, Tabrizi S, et al. Clinical and laboratory predictors of Lassa fever outcome in a dedicated treatment facility in Nigeria: a retrospective, observational cohort study. Lancet Infect Dis. 2018;18(6):684–95. 29523497 10.1016/S1473-3099(18)30121-X PMC5984133

[61] Garry RF. Lassa fever - the road ahead. Nat Rev Microbiol. 2023;21(2):87–96. 36097163 10.1038/s41579-022-00789-8 PMC9466315

[62] Lie KC, Lau CY, Van Vinh CN, West TE, Limmathurotsakul D. Utility of sofa score, management and outcomes of sepsis in Southeast Asia: a multinational multicenter prospective observational study. J Intensive Care. 2018;6:9. 29468069 10.1186/s40560-018-0279-7 PMC5813360

[63] Boissier F, Aissaoui N. Septic cardiomyopathy: diagnosis and management. J Intensive Med. 2022;2(1):8–16. 36789232 10.1016/j.jointm.2021.11.004 PMC9923980

[64] Merx MW, Weber C. Sepsis and the heart. Circulation. 2007;116(7):793–802. 17698745 10.1161/CIRCULATIONAHA.106.678359

[65] Kwong JC, Schwartz KL, Campitelli MA, Chung H, Crowcroft NS, Karnauchow T, et al. Acute myocardial infarction after laboratory-confirmed influenza infection. N Engl J Med. 2018;378(4):345–53. 29365305 10.1056/NEJMoa1702090

[66] Zheng YY, Ma YT, Zhang JY, Xie X. COVID-19 and the cardiovascular system. Nat Rev Cardiol. 2020;17(5):259–60. 32139904 10.1038/s41569-020-0360-5 PMC7095524

[67] Whitehorn J, Yacoub S, Anders KL, Macareo LR, Cassetti MC, Van Nguyen VC, et al. Dengue therapeutics, chemoprophylaxis, and allied tools: state of the art and future directions. PLoS Negl Trop Dis. 2014;8(8):e3025. 25166493 10.1371/journal.pntd.0003025 PMC4148227

[68] Macneil A, Nichol ST, Spiropoulou CF. Hantavirus pulmonary syndrome. Virus Res. 2011;162(1–2):138–47. 21945215 10.1016/j.virusres.2011.09.017

[69] Leligdowicz A, Fischer WA 2nd, Uyeki TM, Fletcher TE, Adhikari NK, Portella G, et al. Ebola virus disease and critical illness. Crit Care. 2016;20(1):217. 27468829 10.1186/s13054-016-1325-2 PMC4965892

[70] Ammirati E, Frigerio M, Adler ED, Basso C, Birnie DH, Brambatti M, et al. Management of acute myocarditis and chronic inflammatory cardiomyopathy: an expert consensus document. Circ Heart Fail. 2020;13(11):e007405. 33176455 10.1161/CIRCHEARTFAILURE.120.007405 PMC7673642

[71] Shi S, Qin M, Shen B, Cai Y, Liu T, Yang F, et al. Association of cardiac injury with mortality in hospitalized patients with COVID-19 in Wuhan. China JAMA Cardiol. 2020;5(7):802–10. 32211816 10.1001/jamacardio.2020.0950 PMC7097841

[72] Matthay MA, Zemans RL. The acute respiratory distress syndrome: pathogenesis and treatment. Annu Rev Pathol. 2011;6:147–63. 20936936 10.1146/annurev-pathol-011110-130158 PMC3108259

[73] Short KR, Kroeze E, Fouchier RAM, Kuiken T. Pathogenesis of influenza-induced acute respiratory distress syndrome. Lancet Infect Dis. 2014;14(1):57–69. 24239327 10.1016/S1473-3099(13)70286-X

[74] Ackermann M, Verleden SE, Kuehnel M, Haverich A, Welte T, Laenger F, et al. Pulmonary vascular endothelialitis, thrombosis, and angiogenesis in COVID-19. N Engl J Med. 2020;383(2):120–8. 32437596 10.1056/NEJMoa2015432 PMC7412750

[75] Schildgen V, van den Hoogen B, Fouchier R, Tripp RA, Alvarez R, Manoha C, et al. Human metapneumovirus: lessons learned over the first decade. Clin Microbiol Rev. 2011;24(4):734–54. 21976607 10.1128/CMR.00015-11 PMC3194831

[76] Duggal A, Pinto R, Rubenfeld G, Fowler RA. Global variability in reported mortality for critical illness during the 2009–10 influenza A (H1N1) pandemic: a systematic review and meta-regression to guide reporting of outcomes during disease outbreaks. PLoS ONE. 2016;11(5):e0155044. 27170999 10.1371/journal.pone.0155044 PMC4865181

[77] Peiris JS, Chu CM, Cheng VC, Chan KS, Hung IF, Poon LL, et al. Clinical progression and viral load in a community outbreak of coronavirus-associated SARS pneumonia: a prospective study. Lancet. 2003;361(9371):1767–72. 12781535 10.1016/S0140-6736(03)13412-5 PMC7112410

[78] Wu C, Chen X, Cai Y, Xia J, Zhou X, Xu S, et al. Risk factors associated with acute respiratory distress syndrome and death in patients with coronavirus disease 2019 pneumonia in Wuhan. China JAMA Intern Med. 2020;180(7):934–43. 32167524 10.1001/jamainternmed.2020.0994 PMC7070509

[79] Tzotzos SJ, Fischer B, Fischer H, Zeitlinger M. Incidence of ARDS and outcomes in hospitalized patients with COVID-19: a global literature survey. Crit Care. 2020;24(1):516. 32825837 10.1186/s13054-020-03240-7 PMC7441837

[80] Influenza Investigators ANZIC, System AMOS, Critical illness due to,. A/H1N1 influenza in pregnant and postpartum women: population based cohort study. BMJ. 2009;2010(340):c1279. 10.1136/bmj.c1279 PMC2841744 20299694

[81] Ahmadian E, Hosseiniyan Khatibi SM, Razi Soofiyani S, Abediazar S, Shoja MM, Ardalan M, et al. COVID-19 and kidney injury: pathophysiology and molecular mechanisms. Rev Med Virol. 2021;31(3):e2176. 33022818 10.1002/rmv.2176 PMC7646060

[82] Burdmann EA, Jha V. Acute kidney injury due to tropical infectious diseases and animal venoms: a tale of 2 continents. Kidney Int. 2017;91(5):1033–46. 28088326 10.1016/j.kint.2016.09.051

[83] Tariq M, Kim DM. Hemorrhagic fever with renal syndrome: literature review, epidemiology, clinical picture and pathogenesis. Infect Chemother. 2022;54(1):1–19. 35384417 10.3947/ic.2021.0148 PMC8987181

[84] Bhasin B, Veitla V, Dawson AZ, Garacci Z, Sturgill D, Ozieh MN, et al. AKI in hospitalized patients with COVID-19 and seasonal influenza: a comparative analysis. Kidney 360. 2021;2(4):619–28. 35373047 10.34067/KID.0007322020 PMC8791326

[85] Ng PY, Ip A, Ng AK, Sin SW, Chan JF, To KK, et al. Risk of acute kidney injury in critically-ill patients with COVID-19 compared with seasonal influenza: a retrospective cohort study. EClinicalMedicine. 2024;70:102535. 38516106 10.1016/j.eclinm.2024.102535 PMC10955633

[86] Chan JF, Lau SK, To KK, Cheng VC, Woo PC, Yuen KY. Middle East respiratory syndrome coronavirus: another zoonotic betacoronavirus causing SARS-like disease. Clin Microbiol Rev. 2015;28(2):465–522. 25810418 10.1128/CMR.00102-14 PMC4402954

[87] Scarpioni R, Valsania T, Albertazzi V, Blanco V, DeAmicis S, Manini A, et al. Acute kidney injury, a common and severe complication in hospitalized patients during the COVID-19 pandemic. J Nephrol. 2021;34(4):1019–24. 34146335 10.1007/s40620-021-01087-x PMC8214067

[88] Chan L, Chaudhary K, Saha A, Chauhan K, Vaid A, Zhao S, et al. AKI in hospitalized patients with COVID-19. J Am Soc Nephrol. 2021;32(1):151–60. 32883700 10.1681/ASN.2020050615 PMC7894657

[89] Ratnayake A, Sarnowski A, Sinclair F, Annear NMP, Banerjee D, Chis SI. The dynamics and outcomes of AKI progression during the COVID-19 pandemic. Nephrology (Carlton). 2024;29(6):325–37. 38549280 10.1111/nep.14297

[90] Rahman A, Niloofa R, Jayarajah U, De Mel S, Abeysuriya V, Seneviratne SL. Hematological abnormalities in COVID-19: a narrative review. Am J Trop Med Hyg. 2021;104(4):1188–201. 33606667 10.4269/ajtmh.20-1536 PMC8045618

[91] Wang Z, Gao X, Miao H, Ma X, Ding R. Understanding COVID-19-associated coagulopathy: from PIC to SIC or DIC. J Intensive Med. 2021;1(1):35–41. 36943814 10.1016/j.jointm.2021.03.002 PMC7997848

[92] Jansen AJG, Spaan T, Low HZ, Di Iorio D, van den Brand J, Tieke M, et al. Influenza-induced thrombocytopenia is dependent on the subtype and sialoglycan receptor and increases with virus pathogenicity. Blood Adv. 2020;4(13):2967–78. 32609845 10.1182/bloodadvances.2020001640 PMC7362372

[93] Raadsen M, Du Toit J, Langerak T, van Bussel B, van Gorp E, Goeijenbier M. Thrombocytopenia in virus infections. J Clin Med. 2021;10(4):877. 33672766 10.3390/jcm10040877 PMC7924611

[94] Yang X, Yang Q, Wang Y, Wu Y, Xu J, Yu Y, et al. Thrombocytopenia and its association with mortality in patients with COVID-19. J Thromb Haemost. 2020;18(6):1469–72. 32302435 10.1111/jth.14848 PMC9906135

[95] Iba T, Levy JH. Thrombosis and thrombocytopenia in COVID-19 and after COVID-19 vaccination. Trends Cardiovasc Med. 2022;32(5):249–56. 35202800 10.1016/j.tcm.2022.02.008 PMC8861143

[96] Popescu NI, Lupu C, Lupu F. Disseminated intravascular coagulation and its immune mechanisms. Blood. 2022;139(13):1973–86. 34428280 10.1182/blood.2020007208 PMC8972096

[97] Marcinkiewicz J, Bryniarski K, Nazimek K. Ebola haemorrhagic fever virus: pathogenesis, immune responses, potential prevention. Folia Med Cracov. 2014;54(3):39–48. 25694094

[98] Koyuncu OO, Hogue IB, Enquist LW. Virus infections in the nervous system. Cell Host Microbe. 2013;13(4):379–93. 23601101 10.1016/j.chom.2013.03.010 PMC3647473

[99] Harapan BN, Yoo HJ. Neurological symptoms, manifestations, and complications associated with severe acute respiratory syndrome coronavirus 2 (SARS-COV-2) and coronavirus disease 19 (COVID-19). J Neurol. 2021;268(9):3059–71. 33486564 10.1007/s00415-021-10406-y PMC7826147

[100] Xu L, Liu J, Lu M, Yang D, Zheng X. Liver injury during highly pathogenic human coronavirus infections. Liver Int. 2020;40(5):998–1004. 32170806 10.1111/liv.14435 PMC7228361

[101] Adams DH, Hubscher SG. Systemic viral infections and collateral damage in the liver. Am J Pathol. 2006;168(4):1057–9. 16565481 10.2353/ajpath.2006.051296 PMC1606546

[102] Zhang C, Shi L, Wang FS. Liver injury in COVID-19: management and challenges. Lancet Gastroenterol Hepatol. 2020;5(5):428–30. 32145190 10.1016/S2468-1253(20)30057-1 PMC7129165

[103] Chau TN, Lee KC, Yao H, Tsang TY, Chow TC, Yeung YC, et al. SARS-associated viral hepatitis caused by a novel coronavirus: report of three cases. Hepatology. 2004;39(2):302–10. 14767982 10.1002/hep.20111 PMC7165792

[104] Alsaad KO, Hajeer AH, Al Balwi M, Al Moaiqel M, Al Oudah N, Al Ajlan A, et al. Histopathology of Middle East respiratory syndrome coronovirus (MERS-CoV) infection - clinicopathological and ultrastructural study. Histopathology. 2018;72(3):516–24. 28858401 10.1111/his.13379 PMC7165512

[105] Assimakopoulos SF, Eleftheriotis G, Lagadinou M, Karamouzos V, Dousdampanis P, Siakallis G, et al. SARS-COV-2-induced viral sepsis: the role of gut barrier dysfunction. Microorganisms. 2022;10(5):1050. 35630492 10.3390/microorganisms10051050 PMC9143860

[106] Sell J, Dolan B. Common gastrointestinal infections. Prim Care. 2018;45(3):519–32. 30115338 10.1016/j.pop.2018.05.008

[107] Takeuchi O, Akira S. Pattern recognition receptors and inflammation. Cell. 2010;140(6):805–20. 20303872 10.1016/j.cell.2010.01.022

[108] Sartorius R, Trovato M, Manco R, D’Apice L, De Berardinis P. Exploiting viral sensing mediated by Toll-like receptors to design innovative vaccines. NPJ Vaccines. 2021;6(1):127. 34711839 10.1038/s41541-021-00391-8 PMC8553822

[109] Kawai T, Akira S. Toll-like receptors and their crosstalk with other innate receptors in infection and immunity. Immunity. 2011;34(5):637–50. 21616434 10.1016/j.immuni.2011.05.006

[110] Tsai YT, Chang SY, Lee CN, Kao CL. Human TLR3 recognizes dengue virus and modulates viral replication in vitro. Cell Microbiol. 2009;11(4):604–15. 19134117 10.1111/j.1462-5822.2008.01277.x

[111] Lester SN, Li K. Toll-like receptors in antiviral innate immunity. J Mol Biol. 2014;426(6):1246–64. 24316048 10.1016/j.jmb.2013.11.024 PMC3943763

[112] Karki R, Kanneganti TD. The ‘cytokine storm’: molecular mechanisms and therapeutic prospects. Trends Immunol. 2021;42(8):681–705. 34217595 10.1016/j.it.2021.06.001 PMC9310545

[113] Yang L, Xie X, Tu Z, Fu J, Xu D, Zhou Y. The signal pathways and treatment of cytokine storm in COVID-19. Signal Transduct Target Ther. 2021;6(1):255. 34234112 10.1038/s41392-021-00679-0 PMC8261820

[114] Park A, Iwasaki A. Type I and type III interferons - induction, signaling, evasion, and application to combat COVID-19. Cell Host Microbe. 2020;27(6):870–8. 32464097 10.1016/j.chom.2020.05.008 PMC7255347

[115] Zheng C. The emerging roles of nod-like receptors in antiviral innate immune signaling pathways. Int J Biol Macromol. 2021;169:407–13. 33347926 10.1016/j.ijbiomac.2020.12.127

[116] Xu J, Gao C, He Y, Fang X, Sun D, Peng Z, et al. NLRC3 expression in macrophage impairs glycolysis and host immune defense by modulating the NF-κB-NFAT5 complex during septic immunosuppression. Mol Ther. 2023;31(1):154–73. 36068919 10.1016/j.ymthe.2022.08.023 PMC9840117

[117] Sun D, Xu J, Zhang W, Song C, Gao C, He Y, et al. Negative regulator NLRC3: its potential role and regulatory mechanism in immune response and immune-related diseases. Front Immunol. 2022;13:1012459. 36341336 10.3389/fimmu.2022.1012459 PMC9630602

[118] Sabbah A, Chang TH, Harnack R, Frohlich V, Tominaga K, Dube PH, et al. Activation of innate immune antiviral responses by NOD2. Nat Immunol. 2009;10(10):1073–80. 19701189 10.1038/ni.1782 PMC2752345

[119] Godkowicz M, Druszczyńska M. NOD1, NOD2, and NLRC5 receptors in antiviral and antimycobacterial immunity. Vaccines (Basel). 2022;10(9):1487. 36146565 10.3390/vaccines10091487 PMC9503463

[120] Yin X, Riva L, Pu Y, Martin-Sancho L, Kanamune J, Yamamoto Y, et al. MDA5 governs the innate immune response to SARS-CoV-2 in lung epithelial cells. Cell Rep. 2021;34(2):108628. 33440148 10.1016/j.celrep.2020.108628 PMC7832566

[121] Wang P, Zhu S, Yang L, Cui S, Pan W, Jackson R, et al. NLRP6 regulates intestinal antiviral innate immunity. Science. 2015;350(6262):826–30. 26494172 10.1126/science.aab3145 PMC4927078

[122] Li X, Deng M, Petrucelli AS, Zhu C, Mo J, Zhang L, et al. Viral DNA binding to NLRC3, an inhibitory nucleic acid sensor, unleashes sting, a cyclic dinucleotide receptor that activates type I interferon. Immunity. 2019;50(3):591-9.e6. 30893587 10.1016/j.immuni.2019.02.009 PMC6469509

[123] Islamuddin M, Mustfa SA, Ullah S, Omer U, Kato K, Parveen S. Innate immune response and inflammasome activation during SARS-CoV-2 infection. Inflammation. 2022;45(5):1849–63. 35953688 10.1007/s10753-022-01651-y PMC9371632

[124] Liu T, Tang L, Tang H, Pu J, Gong S, Fang D, et al. Zika virus infection induces acute kidney injury through activating NLRP3 inflammasome via suppressing BCL-2. Front Immunol. 2019;10:1925. 31474993 10.3389/fimmu.2019.01925 PMC6702322

[125] Onomoto K, Onoguchi K, Yoneyama M. Regulation of RIG-I-like receptor-mediated signaling: interaction between host and viral factors. Cell Mol Immunol. 2021;18(3):539–55. 33462384 10.1038/s41423-020-00602-7 PMC7812568

[126] Rehwinkel J, Gack MU. RIG-I-like receptors: their regulation and roles in rna sensing. Nat Rev Immunol. 2020;20(9):537–51. 32203325 10.1038/s41577-020-0288-3 PMC7094958

[127] Anwar S, Ul Islam K, Azmi MI, Iqbal J. cGAS-STING-mediated sensing pathways in DNA and RNA virus infections: crosstalk with other sensing pathways. Arch Virol. 2021;166(12):3255–68. 34622360 10.1007/s00705-021-05211-x

[128] Han L, Zhuang MW, Deng J, Zheng Y, Zhang J, Nan ML, et al. SARS-CoV-2 ORF9B antagonizes type I and III interferons by targeting multiple components of the RIG-I/MDA-5-MAVS, TLR3-TRIF, and cGAS-STING signaling pathways. J Med Virol. 2021;93(9):5376–89. 33913550 10.1002/jmv.27050 PMC8242602

[129] Zheng Y, Liu Q, Wu Y, Ma L, Zhang Z, Liu T, et al. Zika virus elicits inflammation to evade antiviral response by cleaving cGAS via NS1-caspase-1 axis. EMBO J. 2018;37(18):e99347. 30065070 10.15252/embj.201899347 PMC6138430

[130] Decout A, Katz JD, Venkatraman S, Ablasser A. The cGAS-sting pathway as a therapeutic target in inflammatory diseases. Nat Rev Immunol. 2021;21(9):548–69. 33833439 10.1038/s41577-021-00524-z PMC8029610

[131] Hu H, Tian M, Ding C, Yu S. The C/EBP homologous protein (CHOP) transcription factor functions in endoplasmic reticulum stress-induced apoptosis and microbial infection. Front Immunol. 2018;9:3083. 30662442 10.3389/fimmu.2018.03083 PMC6328441

[132] Gong T, Wang QD, Loughran PA, Li YH, Scott MJ, Billiar TR, et al. Mechanism of lactic acidemia-promoted pulmonary endothelial cells death in sepsis: role for CIRP-ZBP1-PANoptosis pathway. Mil Med Res. 2024;11(1):71. 39465383 10.1186/s40779-024-00574-z PMC11514876

[133] Banerjee A, Czinn SJ, Reiter RJ, Blanchard TG. Crosstalk between endoplasmic reticulum stress and anti-viral activities: a novel therapeutic target for COVID-19. Life Sci. 2020;255:117842. 32454157 10.1016/j.lfs.2020.117842 PMC7245231

[134] Schneider A, Kurz S, Manske K, Janas M, Heikenwälder M, Misgeld T, et al. Single organelle analysis to characterize mitochondrial function and crosstalk during viral infection. Sci Rep. 2019;9(1):8492. 31186476 10.1038/s41598-019-44922-9 PMC6560178

[135] Shang C, Liu Z, Zhu Y, Lu J, Ge C, Zhang C, et al. SARS-CoV-2 causes mitochondrial dysfunction and mitophagy impairment. Front Microbiol. 2021;12:780768. 35069483 10.3389/fmicb.2021.780768 PMC8770829

[136] Loncke J, Kaasik A, Bezprozvanny I, Parys JB, Kerkhofs M, Bultynck G. Balancing er-mitochondrial Ca^2+^ fluxes in health and disease. Trends Cell Biol. 2021;31(7):598–612. 33678551 10.1016/j.tcb.2021.02.003 PMC8195822

[137] Arulkumaran N, Deutschman CS, Pinsky MR, Zuckerbraun B, Schumacker PT, Gomez H, et al. Mitochondrial function in sepsis. Shock. 2016;45(3):271–81. 26871665 10.1097/SHK.0000000000000463 PMC4755359

[138] de Miranda FS, Claudio L, de Almeida DSM, Nunes JB, Barauna VG, Luiz WB, et al. Cell-free nuclear and mitochondrial DNA as potential biomarkers for assessing sepsis severity. Biomedicines. 2024;12(5):933. 38790895 10.3390/biomedicines12050933 PMC11117867

[139] Mao JY, Li DK, Zhang HM, Wang XT, Liu DW. Plasma mitochondrial DNA levels are associated with acute lung injury and mortality in septic patients. BMC Pulm Med. 2021;21(1):66. 33632166 10.1186/s12890-021-01437-2 PMC7905766

[140] Zhang YY, Ning BT. Signaling pathways and intervention therapies in sepsis. Signal Transduct Target Ther. 2021;6(1):407. 34824200 10.1038/s41392-021-00816-9 PMC8613465

[141] Thaker SK, Ch’ng J, Christofk HR. Viral hijacking of cellular metabolism. BMC Biol. 2019;17(1):59. 31319842 10.1186/s12915-019-0678-9 PMC6637495

[142] Kangussu LM, Costa VV, Olivon VC, Queiroz-Junior CM, Gondim ANS, Melo MB, et al. Dengue virus infection induces inflammation and oxidative stress on the heart. Heart. 2022;108(5):388–96. 34049953 10.1136/heartjnl-2020-318912

[143] Ammer-Herrmenau C, Kulkarni U, Andreas N, Ungelenk M, Ravens S, Hübner C, et al. Sepsis induces long-lasting impairments in CD4^+^ T-cell responses despite rapid numerical recovery of T-lymphocyte populations. PLoS One. 2019;14(2):e0211716. 30730978 10.1371/journal.pone.0211716 PMC6366777

[144] Bellesi S, Metafuni E, Hohaus S, Maiolo E, Marchionni F, D’Innocenzo S, et al. Increased CD95 (Fas) and PD-1 expression in peripheral blood T lymphocytes in COVID-19 patients. Br J Haematol. 2020;191(2):207–11. 32679621 10.1111/bjh.17034 PMC7405050

[145] Monneret G, Venet F. A rapidly progressing lymphocyte exhaustion after severe sepsis. Crit Care. 2012;16(4):140. 22824381 10.1186/cc11416 PMC3580699

[146] Zhang T, Yin C, Boyd DF, Quarato G, Ingram JP, Shubina M, et al. Influenza virus Z-RNAs induce ZBP1-mediated necroptosis. Cell. 2020;180(6):1115-29.e13. 32200799 10.1016/j.cell.2020.02.050 PMC7153753

[147] Li S, Zhang Y, Guan Z, Ye M, Li H, You M, et al. SARS-CoV-2 Z-RNA activates the ZBP1-RIPK3 pathway to promote virus-induced inflammatory responses. Cell Res. 2023;33(3):201–14. 36650286 10.1038/s41422-022-00775-y PMC9844202

[148] Wu MF, Chen ST, Yang AH, Lin WW, Lin YL, Chen NJ, et al. CLEC5A is critical for dengue virus-induced inflammasome activation in human macrophages. Blood. 2013;121(1):95–106. 23152543 10.1182/blood-2012-05-430090

[149] Qu M, Wang Y, Qiu Z, Zhu S, Guo K, Chen W, et al. Necroptosis, pyroptosis, ferroptosis in sepsis and treatment. Shock. 2022;57(6):161–71. 35759299 10.1097/SHK.0000000000001936

[150] Verdonck S, Nemegeer J, Vandenabeele P, Maelfait J. Viral manipulation of host cell necroptosis and pyroptosis. Trends Microbiol. 2022;30(6):593–605. 34933805 10.1016/j.tim.2021.11.011

[151] Pan P, Shen M, Yu Z, Ge W, Chen K, Tian M, et al. SARS-CoV-2 N protein promotes NLRP3 inflammasome activation to induce hyperinflammation. Nat Commun. 2021;12(1):4664. 34341353 10.1038/s41467-021-25015-6 PMC8329225

[152] Imre G. Cell death signalling in virus infection. Cell Signal. 2020;76:109772. 32931899 10.1016/j.cellsig.2020.109772 PMC7486881

[153] Chen YJ, Wang SF, Weng IC, Hong MH, Lo TH, Jan JT, et al. Galectin-3 enhances avian H5N1 influenza A virus-induced pulmonary inflammation by promoting NLRP3 inflammasome activation. Am J Pathol. 2018;188(4):1031–42. 29366678 10.1016/j.ajpath.2017.12.014

[154] Dixon SJ, Olzmann JA. The cell biology of ferroptosis. Nat Rev Mol Cell Biol. 2024;25(6):424–42. 38366038 10.1038/s41580-024-00703-5 PMC12187608

[155] Wang J, Zhu J, Ren S, Zhang Z, Niu K, Li H, et al. The role of ferroptosis in virus infections. Front Microbiol. 2023;14:1279655. 38075884 10.3389/fmicb.2023.1279655 PMC10706002

[156] Yu Y, Yan Y, Niu F, Wang Y, Chen X, Su G, et al. Ferroptosis: a cell death connecting oxidative stress, inflammation and cardiovascular diseases. Cell Death Discov. 2021;7(1):193. 34312370 10.1038/s41420-021-00579-w PMC8313570

[157] Nguyen LN, Kanneganti TD. Panoptosis in viral infection: the missing puzzle piece in the cell death field. J Mol Biol. 2022;434(4):167249. 34537233 10.1016/j.jmb.2021.167249 PMC8444475

[158] Lee S, Karki R, Wang Y, Nguyen LN, Kalathur RC, Kanneganti TD. AIM2 forms a complex with pyrin and ZBP1 to drive panoptosis and host defence. Nature. 2021;597(7876):415–9. 34471287 10.1038/s41586-021-03875-8 PMC8603942

[159] Parzych KR, Klionsky DJ. An overview of autophagy: morphology, mechanism, and regulation. Antioxid Redox Signal. 2014;20(3):460–73. 23725295 10.1089/ars.2013.5371 PMC3894687

[160] Qiu P, Liu Y, Zhang J. Review: the role and mechanisms of macrophage autophagy in sepsis. Inflammation. 2019;42(1):6–19. 30194660 10.1007/s10753-018-0890-8

[161] Miao G, Zhao H, Li Y, Ji M, Chen Y, Shi Y, et al. ORF3A of the COVID-19 virus SARS-CoV-2 blocks hops complex-mediated assembly of the snare complex required for autolysosome formation. Dev Cell. 2021;56(4):427-42.e5. 33422265 10.1016/j.devcel.2020.12.010 PMC7832235

[162] Hoenigsperger H, Koepke L, Acharya D, Hunszinger V, Freisem D, Grenzner A, et al. CSNK2 suppresses autophagy by activating FLN-NHL-containing trim proteins. Autophagy. 2024;20(5):994–1014. 37938186 10.1080/15548627.2023.2281128 PMC11135829

[163] Chen T, Tu S, Ding L, Jin M, Chen H, Zhou H. The role of autophagy in viral infections. J Biomed Sci. 2023;30(1):5. 36653801 10.1186/s12929-023-00899-2 PMC9846652

[164] Jassey A, Jackson WT. Viruses and autophagy: bend, but don’t break. Nat Rev Microbiol. 2024;22(5):309–21. 38102460 10.1038/s41579-023-00995-y

[165] Yuan S, Jiang SC, Zhang ZW, Fu YF, Hu J, Li ZL. Quantification of cytokine storms during virus infections. Front Immunol. 2021;12:659419. 34079547 10.3389/fimmu.2021.659419 PMC8165266

[166] Nanaware N, Banerjee A, Mullick Bagchi S, Bagchi P, Mukherjee A. Dengue virus infection: a tale of viral exploitations and host responses. Viruses. 2021;13(10):1967. 34696397 10.3390/v13101967 PMC8541669

[167] de Vries F, Huckriede J, Wichapong K, Reutelingsperger C, Nicolaes GAF. The role of extracellular histones in COVID-19. J Intern Med. 2023;293(3):275–92. 36382685 10.1111/joim.13585 PMC10108027

[168] Li X, Ye Y, Peng K, Zeng Z, Chen L, Zeng Y. Histones: the critical players in innate immunity. Front Immunol. 2022;13:1030610. 36479112 10.3389/fimmu.2022.1030610 PMC9720293

[169] Ligi D, Lo Sasso B, Giglio RV, Maniscalco R, DellaFranca C, Agnello L, et al. Circulating histones contribute to monocyte and MDW alterations as common mediators in classical and COVID-19 sepsis. Crit Care. 2022;26(1):260. 36042461 10.1186/s13054-022-04138-2 PMC9424804

[170] Ligi D, Giglio RV, Henry BM, Lippi G, Ciaccio M, Plebani M, et al. What is the impact of circulating histones in COVID-19: a systematic review. Clin Chem Lab Med. 2022;60(10):1506–17. 35852070 10.1515/cclm-2022-0574

[171] Ligi D, Maniscalco R, Plebani M, Lippi G, Mannello F. Do circulating histones represent the missing link among COVID-19 infection and multiorgan injuries, microvascular coagulopathy and systemic hyperinflammation?. J Clin Med. 2022;11(7):1800. 35407410 10.3390/jcm11071800 PMC8999947

[172] Middleton EA, He XY, Denorme F, Campbell RA, Ng D, Salvatore SP, et al. Neutrophil extracellular traps contribute to immunothrombosis in COVID-19 acute respiratory distress syndrome. Blood. 2020;136(10):1169–79. 32597954 10.1182/blood.2020007008 PMC7472714

[173] Ashar HK, Mueller NC, Rudd JM, Snider TA, Achanta M, Prasanthi M, et al. The role of extracellular histones in influenza virus pathogenesis. Am J Pathol. 2018;188(1):135–48. 29107075 10.1016/j.ajpath.2017.09.014 PMC5745522

[174] Papayannopoulos V. Neutrophil extracellular traps in immunity and disease. Nat Rev Immunol. 2018;18(2):134–47. 28990587 10.1038/nri.2017.105

[175] Zuo Y, Yalavarthi S, Shi H, Gockman K, Zuo M, Madison JA, et al. Neutrophil extracellular traps in COVID-19. JCI Insight. 2020;5(11):e138999. 32329756 10.1172/jci.insight.138999 PMC7308057

[176] Maucourant C, Filipovic I, Ponzetta A, Aleman S, Cornillet M, Hertwig L, et al. Natural killer cell immunotypes related to COVID-19 disease severity. Sci Immunol. 2020;5(50):eabd6832. 32826343 10.1126/sciimmunol.abd6832 PMC7665314

[177] Björkström NK, Strunz B, Ljunggren HG. Natural killer cells in antiviral immunity. Nat Rev Immunol. 2022;22(2):112–23. 34117484 10.1038/s41577-021-00558-3 PMC8194386

[178] Takahashi Y, Onodera T, Adachi Y, Ato M. Adaptive B cell responses to influenza virus infection in the lung. Viral Immunol. 2017;30(6):431–7. 28661720 10.1089/vim.2017.0025

[179] Upasani V, Vo HTM, Auerswald H, Laurent D, Heng S, Duong V, et al. Direct infection of B cells by dengue virus modulates B cell responses in a Cambodian pediatric cohort. Front Immunol. 2020;11:594813. 33643283 10.3389/fimmu.2020.594813 PMC7907177

[180] Ma C, Liu H, Yang S, Li H, Liao X, Kang Y. The emerging roles and therapeutic potential of B cells in sepsis. Front Pharmacol. 2022;13:1034667. 36425582 10.3389/fphar.2022.1034667 PMC9679374

[181] Dine E, Reed EH, Toettcher JE. Positive feedback between the T cell kinase ZAP70 and its substrate lat acts as a clustering-dependent signaling switch. Cell Rep. 2021;35(12):109280. 34161759 10.1016/j.celrep.2021.109280 PMC8292983

[182] Hillaire ML, Rimmelzwaan GF, Kreijtz JH. Clearance of influenza virus infections by T cells: risk of collateral damage?. Curr Opin Virol. 2013;3(4):430–7. 23721864 10.1016/j.coviro.2013.05.002

[183] Moss P. The T cell immune response against SARS-CoV-2. Nat Immunol. 2022;23(2):186–93. 35105982 10.1038/s41590-021-01122-w

[184] Rodrigues TS, de Sá KSG, Ishimoto AY, Becerra A, Oliveira S, Almeida L, et al. Inflammasomes are activated in response to SARS-CoV-2 infection and are associated with COVID-19 severity in patients. J Exp Med. 2021;218(3):e20201707. 33231615 10.1084/jem.20201707 PMC7684031

[185] Nothelfer K, Sansonetti PJ, Phalipon A. Pathogen manipulation of B cells: the best defence is a good offence. Nat Rev Microbiol. 2015;13(3):173–84. 25659322 10.1038/nrmicro3415

[186] Upasani V, Rodenhuis-Zybert I, Cantaert T. Antibody-independent functions of B cells during viral infections. PLoS Pathog. 2021;17(7):e1009708. 34293057 10.1371/journal.ppat.1009708 PMC8297758

[187] Lam JH, Smith FL, Baumgarth N. B cell activation and response regulation during viral infections. Viral Immunol. 2020;33(4):294–306. 32326852 10.1089/vim.2019.0207 PMC7247032

[188] Rha MS, Shin EC. Activation or exhaustion of CD8^+^ T cells in patients with COVID-19. Cell Mol Immunol. 2021;18(10):2325–33. 34413488 10.1038/s41423-021-00750-4 PMC8374113

[189] Tian W, Zhang N, Jin R, Feng Y, Wang S, Gao S, et al. Immune suppression in the early stage of COVID-19 disease. Nat Commun. 2020;11(1):5859. 33203833 10.1038/s41467-020-19706-9 PMC7673112

[190] Xu J, Yang X, Yang L, Zou X, Wang Y, Wu Y, et al. Clinical course and predictors of 60-day mortality in 239 critically ill patients with COVID-19: a multicenter retrospective study from Wuhan, China. Crit Care. 2020;24(1):394. 32631393 10.1186/s13054-020-03098-9 PMC7336107

[191] Veglia F, Sanseviero E, Gabrilovich DI. Myeloid-derived suppressor cells in the era of increasing myeloid cell diversity. Nat Rev Immunol. 2021;21(8):485–98. 33526920 10.1038/s41577-020-00490-y PMC7849958

[192] Kahan SM, Wherry EJ, Zajac AJ. T cell exhaustion during persistent viral infections. Virology. 2015;479–480:180–93. 25620767 10.1016/j.virol.2014.12.033 PMC4424083

[193] Malavige GN, Ogg GS. Pathogenesis of vascular leak in dengue virus infection. Immunology. 2017;151(3):261–9. 28437586 10.1111/imm.12748 PMC5461104

[194] Gavrilovskaya IN, Gorbunova EE, Mackow NA, Mackow ER. Hantaviruses direct endothelial cell permeability by sensitizing cells to the vascular permeability factor VEGF, while angiopoietin 1 and sphingosine 1-phosphate inhibit hantavirus-directed permeability. J Virol. 2008;82(12):5797–806. 18367532 10.1128/JVI.02397-07 PMC2395149

[195] Sugiyama MG, Armstrong SM, Wang C, Hwang D, Leong-Poi H, Advani A, et al. The TIE2-agonist vasculotide rescues mice from influenza virus infection. Sci Rep. 2015;5:11030. 26046800 10.1038/srep11030 PMC4457136

[196] Barrett TJ, Cornwell M, Myndzar K, Rolling CC, Xia Y, Drenkova K, et al. Platelets amplify endotheliopathy in COVID-19. Sci Adv. 2021;7(37):eabh2434. 34516880 10.1126/sciadv.abh2434 PMC8442885

[197] Singh A, Bisht P, Bhattacharya S, Guchhait P. Role of platelet cytokines in dengue virus infection. Front Cell Infect Microbiol. 2020;10:561366. 33102253 10.3389/fcimb.2020.561366 PMC7554584

[198] Mandel J, Casari M, Stepanyan M, Martyanov A, Deppermann C. Beyond hemostasis: platelet innate immune interactions and thromboinflammation. Int J Mol Sci. 2022;23(7):3868. 35409226 10.3390/ijms23073868 PMC8998935

[199] Kim SJ, Carestia A, McDonald B, Zucoloto AZ, Grosjean H, Davis RP, et al. Platelet-mediated net release amplifies coagulopathy and drives lung pathology during severe influenza infection. Front Immunol. 2021;12:772859. 34858432 10.3389/fimmu.2021.772859 PMC8632260

[200] Chao CH, Wu WC, Lai YC, Tsai PJ, Perng GC, Lin YS, et al. Dengue virus nonstructural protein 1 activates platelets via Toll-like receptor 4, leading to thrombocytopenia and hemorrhage. PLoS Pathog. 2019;15(4):e1007625. 31009511 10.1371/journal.ppat.1007625 PMC6497319

[201] Fang L, Yu S, Tian X, Fu W, Su L, Chen Z, et al. Severe fever with thrombocytopenia syndrome virus replicates in platelets and enhances platelet activation. J Thromb Haemost. 2023;21(5):1336–51. 36792011 10.1016/j.jtha.2023.02.006

[202] Karasu E, Nilsson B, Köhl J, Lambris JD, Huber-Lang M. Targeting complement pathways in polytrauma- and sepsis-induced multiple-organ dysfunction. Front Immunol. 2019;10:543. 30949180 10.3389/fimmu.2019.00543 PMC6437067

[203] Afzali B, Noris M, Lambrecht BN, Kemper C. The state of complement in COVID-19. Nat Rev Immunol. 2022;22(2):77–84. 34912108 10.1038/s41577-021-00665-1 PMC8672651

[204] Jiang Y, Zhao G, Song N, Li P, Chen Y, Guo Y, et al. Blockade of the C5A–C5AR axis alleviates lung damage in HDPP4-transgenic mice infected with MERS-CoV. Emerg Microbes Infect. 2018;7(1):77. 29691378 10.1038/s41426-018-0063-8 PMC5915580

[205] Carr JM, Cabezas-Falcon S, Dubowsky JG, Hulme-Jones J, Gordon DL. Dengue virus and the complement alternative pathway. FEBS Lett. 2020;594(16):2543–55. 31943152 10.1002/1873-3468.13730

[206] Cai X, Panicker SR, Biswas I, Giri H, Rezaie AR. Protective role of activated protein C against viral mimetic poly(i:C)-induced inflammation. Thromb Haemost. 2021;121(11):1448–63. 33706396 10.1055/s-0041-1726093 PMC8433266

[207] Mackman N, Grover SP, Antoniak S. Tissue factor expression, extracellular vesicles, and thrombosis after infection with the respiratory viruses influenza A virus and coronavirus. J Thromb Haemost. 2021;19(11):2652–8. 34418279 10.1111/jth.15509 PMC9770926

[208] Schouten M, Sluijs KF, Gerlitz B, Grinnell BW, Roelofs JJ, Levi MM, et al. Activated protein c ameliorates coagulopathy but does not influence outcome in lethal H1N1 influenza: a controlled laboratory study. Crit Care. 2010;14(2):R65. 20398279 10.1186/cc8964 PMC2887187

[209] Sharma S, Ursery LT, Bharathi V, Miles SD, Williams WA, Elzawam AZ, et al. APC-PAR1-R46 signaling limits CXCL1 expression during poly IC-induced airway inflammation in mice. J Thromb Haemost. 2023;21(11):3279–82. 37634652 10.1016/j.jtha.2023.08.018

[210] Zhang Y, Bharathi V, Dokoshi T, de Anda J, Ursery LT, Kulkarni NN, et al. Viral afterlife: SARS-CoV-2 as a reservoir of immunomimetic peptides that reassemble into proinflammatory supramolecular complexes. Proc Natl Acad Sci U S A. 2024;121(6):e2300644120. 38306481 10.1073/pnas.2300644120 PMC10861912

[211] Rello J, Valenzuela-Sánchez F, Ruiz-Rodriguez M, Moyano S. Sepsis: a review of advances in management. Adv Ther. 2017;34(11):2393–411. 29022217 10.1007/s12325-017-0622-8 PMC5702377

[212] Guzman MG, Harris E. Dengue. Lancet. 2015;385(9966):453–65. 25230594 10.1016/S0140-6736(14)60572-9

[213] Zhang H, Xu Y, Huang X, Yang S, Li R, Wu Y, et al. Extracorporeal membrane oxygenation in adult patients with sepsis and septic shock: why, how, when, and for whom. J Intensive Med. 2024;4(1):62–72. 38263962 10.1016/j.jointm.2023.07.001 PMC10800772

[214] Ling RR, Ramanathan K, Poon WH, Tan CS, Brechot N, Brodie D, et al. Venoarterial extracorporeal membrane oxygenation as mechanical circulatory support in adult septic shock: a systematic review and meta-analysis with individual participant data meta-regression analysis. Crit Care. 2021;25(1):246. 34261492 10.1186/s13054-021-03668-5 PMC8278703

[215] Ma X, Liang M, Ding M, Liu W, Ma H, Zhou X, et al. Extracorporeal membrane oxygenation (ECMO) in critically ill patients with coronavirus disease 2019 (COVID-19) pneumonia and acute respiratory distress syndrome (ARDS). Med Sci Monit. 2020;26:e925364. 32759887 10.12659/MSM.925364 PMC7430351

[216] Lameire N, Vanmassenhove J. Timing of dialysis in sepsis and acute respiratory distress syndrome. Am J Respir Crit Care Med. 2018;198(1):4–5. 29394089 10.1164/rccm.201801-0129ED

[217] Monteil V, Kwon H, Prado P, Hagelkrüys A, Wimmer RA, Stahl M, et al. Inhibition of SARS-CoV-2 infections in engineered human tissues using clinical-grade soluble human ACE2. Cell. 2020;181(4):905-13.e7. 32333836 10.1016/j.cell.2020.04.004 PMC7181998

[218] Zoufaly A, Poglitsch M, Aberle JH, Hoepler W, Seitz T, Traugott M, et al. Human recombinant soluble ACE2 in severe COVID-19. Lancet Respir Med. 2020;8(11):1154–8. 33131609 10.1016/S2213-2600(20)30418-5 PMC7515587

[219] Lu L, Su S, Yang H, Jiang S. Antivirals with common targets against highly pathogenic viruses. Cell. 2021;184(6):1604–20. 33740455 10.1016/j.cell.2021.02.013

[220] Egorova A, Kazakova E, Jahn B, Ekins S, Makarov V, Schmidtke M. Novel pleconaril derivatives: Influence of substituents in the isoxazole and phenyl rings on the antiviral activity against enteroviruses. Eur J Med Chem. 2020;188:112007. 31881489 10.1016/j.ejmech.2019.112007 PMC7002245

[221] Abzug MJ, Michaels MG, Wald E, Jacobs RF, Romero JR, Sánchez PJ, et al. A randomized, double-blind, placebo-controlled trial of pleconaril for the treatment of neonates with enterovirus sepsis. J Pediatric Infect Dis Soc. 2016;5(1):53–62. 26407253 10.1093/jpids/piv015 PMC4765488

[222] Wec AZ, Herbert AS, Murin CD, Nyakatura EK, Abelson DM, Fels JM, et al. Antibodies from a human survivor define sites of vulnerability for broad protection against ebolaviruses. Cell. 2017;169(5):878-90.e15. 28525755 10.1016/j.cell.2017.04.037 PMC5808922

[223] Lu L, Liu Q, Zhu Y, Chan KH, Qin L, Li Y, et al. Structure-based discovery of Middle East respiratory syndrome coronavirus fusion inhibitor. Nat Commun. 2014;5:3067. 24473083 10.1038/ncomms4067 PMC7091805

[224] Xia S, Lan Q, Zhu Y, Wang C, Xu W, Li Y, et al. Structural and functional basis for pan-CoV fusion inhibitors against SARS-CoV-2 and its variants with preclinical evaluation. Signal Transduct Target Ther. 2021;6(1):288. 34326308 10.1038/s41392-021-00712-2 PMC8320318

[225] Koszalka P, Tilmanis D, Hurt AC. Influenza antivirals currently in late-phase clinical trial. Influenza Other Respir Viruses. 2017;11(3):240–6. 28146320 10.1111/irv.12446 PMC5410715

[226] Miao G, Peng H, Tang H, Liu Y, Zheng X, Liu B, et al. Antiviral efficacy of selective estrogen receptor modulators against SARS-CoV-2 infection in vitro and in vivo reveals bazedoxifene acetate as an entry inhibitor. J Med Virol. 2022;94(10):4809–19. 35733297 10.1002/jmv.27951 PMC9350378

[227] Li H, Jiang XM, Cui N, Yuan C, Zhang SF, Lu QB, et al. Clinical effect and antiviral mechanism of T-705 in treating severe fever with thrombocytopenia syndrome. Signal Transduct Target Ther. 2021;6(1):145. 33859168 10.1038/s41392-021-00541-3 PMC8050330

[228] Karim M, Lo CW, Einav S. Preparing for the next viral threat with broad-spectrum antivirals. J Clin Invest. 2023;133(11):e170236. 37259914 10.1172/JCI170236 PMC10232003

[229] Beaucourt S, Vignuzzi M. Ribavirin: a drug active against many viruses with multiple effects on virus replication and propagation. Molecular basis of ribavirin resistance. Curr Opin Virol. 2014;8:10–5. 24846716 10.1016/j.coviro.2014.04.011 PMC7102760

[230] Zhang Y, Sun Y, Xie Y, Shang W, Wang Z, Jiang H, et al. A viral RNA-dependent rna polymerase inhibitor VV116 broadly inhibits human coronaviruses and has synergistic potency with 3CLPRO inhibitor nirmatrelvir. Signal Transduct Target Ther. 2023;8(1):360. 37735468 10.1038/s41392-023-01587-1 PMC10514301

[231] Harris E. FDA grants full approval to paxlovid, COVID-19 antiviral treatment. JAMA. 2023;329(24):2118. 37285173 10.1001/jama.2023.9925

[232] Shahryari A, Saghaeian Jazi M, Mohammadi S, Razavi Nikoo H, Nazari Z, Hosseini ES, et al. Development and clinical translation of approved gene therapy products for genetic disorders. Front Genet. 2019;10:868. 31608113 10.3389/fgene.2019.00868 PMC6773888

[233] Campochiaro C, Della-Torre E, Cavalli G, De Luca G, Ripa M, Boffini N, et al. Efficacy and safety of tocilizumab in severe COVID-19 patients: a single-centre retrospective cohort study. Eur J Intern Med. 2020;76:43–9. 32482597 10.1016/j.ejim.2020.05.021 PMC7242960

[234] Cavalli G, De Luca G, Campochiaro C, Della-Torre E, Ripa M, Canetti D, et al. Interleukin-1 blockade with high-dose anakinra in patients with COVID-19, acute respiratory distress syndrome, and hyperinflammation: a retrospective cohort study. Lancet Rheumatol. 2020;2(6):e325–31. 32501454 10.1016/S2665-9913(20)30127-2 PMC7252085

[235] Cao B, Wang Y, Wen D, Liu W, Wang J, Fan G, et al. A trial of lopinavir-ritonavir in adults hospitalized with severe COVID-19. N Engl J Med. 2020;382(19):1787–99. 32187464 10.1056/NEJMoa2001282 PMC7121492

[236] RECOVERY Collaborative Group. Lopinavir-ritonavir in patients admitted to hospital with COVID-19 (recovery): a randomised, controlled, open-label, platform trial. Lancet. 2020;396(10259):1345–52. 33031764 10.1016/S0140-6736(20)32013-4 PMC7535623

[237] Marjuki H, Mishin VP, Chesnokov AP, De La Cruz JA, Fry AM, Villanueva J, et al. An investigational antiviral drug, DAS181, effectively inhibits replication of zoonotic influenza A virus subtype H7N9 and protects mice from lethality. J Infect Dis. 2014;210(3):435–40. 24569063 10.1093/infdis/jiu105 PMC4091581

[238] Markham A. Fostemsavir: first approval. Drugs. 2020;80(14):1485–90. 32852743 10.1007/s40265-020-01386-w

[239] Lataillade M, Lalezari JP, Kozal M, Aberg JA, Pialoux G, Cahn P, et al. Safety and efficacy of the HIV-1 attachment inhibitor prodrug fostemsavir in heavily treatment-experienced individuals: week 96 results of the phase 3 brighte study. Lancet HIV. 2020;7(11):e740–51. 33128903 10.1016/S2352-3018(20)30240-X

[240] Channappanavar R, Lu L, Xia S, Du L, Meyerholz DK, Perlman S, et al. Protective effect of intranasal regimens containing peptidic Middle East respiratory syndrome coronavirus fusion inhibitor against MERS-CoV infection. J Infect Dis. 2015;212(12):1894–903. 26164863 10.1093/infdis/jiv325 PMC4655857

[241] Di Stefano L, Ogburn EL, Ram M, Scharfstein DO, Li T, et al. Hydroxychloroquine/chloroquine for the treatment of hospitalized patients with COVID-19: an individual participant data meta-analysis. PLoS One. 2022;17(9):e0273526. 36173983 10.1371/journal.pone.0273526 PMC9521809

[242] Hong H, Friedland A, Hu M, Anstrom KJ, Halabi S, McKinnon JE, et al. Safety and efficacy of hydroxychloroquine as prophylactic against COVID-19 in healthcare workers: a meta-analysis of randomised clinical trials. BMJ Open. 2023;13(6):e065305. 37328184 10.1136/bmjopen-2022-065305 PMC10276967

[243] Axfors C, Schmitt AM, Janiaud P, Van’t Hooft J, Abd-Elsalam S, Abdo EF, et al. Mortality outcomes with hydroxychloroquine and chloroquine in COVID-19 from an international collaborative meta-analysis of randomized trials. Nat Commun. 2021;12(1):2349. 33859192 10.1038/s41467-021-22446-z PMC8050319

[244] Liang C, Tian L, Liu Y, Hui N, Qiao G, Li H, et al. A promising antiviral candidate drug for the COVID-19 pandemic: a mini-review of remdesivir. Eur J Med Chem. 2020;201:112527. 32563812 10.1016/j.ejmech.2020.112527 PMC7834743

[245] Marocco R, Del Borgo C, Tortellini E, Garattini S, Carraro A, Di Trento D, et al. Use of remdesivir in patients with SARS-CoV-2 pneumonia in a real-life setting during the second and third COVID-19 epidemic waves. Viruses. 2023;15(4):947. 37112927 10.3390/v15040947 PMC10143300

[246] Gottlieb RL, Vaca CE, Paredes R, Mera J, Webb BJ, Perez G, et al. Early remdesivir to prevent progression to severe COVID-19 in outpatients. N Engl J Med. 2022;386(4):305–15. 34937145 10.1056/NEJMoa2116846 PMC8757570

[247] Chen MP, Jiang DX, Rang JX, Zhuo HB, Zhou ZG. Comparison of azvudine, molnupiravir, and nirmatrelvir/ritonavir in adult patients with mild-to-moderate COVID-19: a retrospective cohort study. Sci Rep. 2024;14(1):3318. 38337014 10.1038/s41598-024-53862-y PMC10858188

[248] Hammond J, Leister-Tebbe H, Gardner A, Abreu P, Bao W, Wisemandle W, et al. Oral nirmatrelvir for high-risk, nonhospitalized adults with COVID-19. N Engl J Med. 2022;386(15):1397–408. 35172054 10.1056/NEJMoa2118542 PMC8908851

[249] Huang C, Shuai H, Qiao J, Hou Y, Zeng R, Xia A, et al. A new generation M^pro^ inhibitor with potent activity against SARS-CoV-2 omicron variants. Signal Transduct Target Ther. 2023;8(1):128. 36928316 10.1038/s41392-023-01392-w PMC10018608

[250] Sasaki M, Tabata K, Kishimoto M, Itakura Y, Kobayashi H, Ariizumi T, et al. S-217622, a SARS-CoV-2 main protease inhibitor, decreases viral load and ameliorates COVID-19 severity in hamsters. Sci Transl Med. 2023;15(679):eabq4064. 36327352 10.1126/scitranslmed.abq4064 PMC9765455

[251] Çakmak R, Yüce S, Ay M, Uyar MH, Kılıç M, Bektaş M. Intravenous high-dose anakinra drops venous thrombosis and acute coronary syndrome in severe and critical COVID-19 patients: a propensity score matched study. Sci Rep. 2024;14(1):12369. 38811592 10.1038/s41598-024-62079-y PMC11137068

[252] Menzella F, Fontana M, Salvarani C, Massari M, Ruggiero P, Scelfo C, et al. Efficacy of tocilizumab in patients with COVID-19 ARDS undergoing noninvasive ventilation. Crit Care. 2020;24(1):589. 32993751 10.1186/s13054-020-03306-6 PMC7523258

[253] Jhan MK, HuangFu WC, Chen YF, Kao JC, Tsai TT, Ho MR, et al. Anti-TNF-α restricts dengue virus-induced neuropathy. J Leukoc Biol. 2018;104(5):961–8. 30044892 10.1002/JLB.MA1217-484R

[254] Sundén-Cullberg J, Chen P, Häbel H, Skorup P, Janols H, Rasmuson J, et al. Anakinra or tocilizumab in patients admitted to hospital with severe COVID-19 at high risk of deterioration (IMMCoVA): a randomized, controlled, open-label trial. PLoS One. 2023;18(12):e0295838. 38157348 10.1371/journal.pone.0295838 PMC10756513

[255] Rossotti R, Travi G, Ughi N, Corradin M, Baiguera C, Fumagalli R, et al. Safety and efficacy of anti-IL6-receptor tocilizumab use in severe and critical patients affected by coronavirus disease 2019: a comparative analysis. J Infect. 2020;81(4):e11–7. 32652164 10.1016/j.jinf.2020.07.008 PMC7345400

[256] Kimmig LM, Wu D, Gold M, Pettit NN, Pitrak D, Mueller J, et al. IL-6 inhibition in critically ill COVID-19 patients is associated with increased secondary infections. Front Med (Lausanne). 2020;7:583897. 33195334 10.3389/fmed.2020.583897 PMC7655919

[257] Wu W, Arunagiri V, Do-Umehara HC, Chen C, Gu S, Biswas I, et al. MIZ1 represses type I interferon production and limits viral clearance during influenza A virus infection. Sci Signal. 2024;17(831):eadg7867. 38593156 10.1126/scisignal.adg7867 PMC11182629

[258] Wang N, Zhan Y, Zhu L, Hou Z, Liu F, Song P, et al. Retrospective multicenter cohort study shows early interferon therapy is associated with favorable clinical responses in COVID-19 patients. Cell Host Microbe. 2020;28(3):455-64.e2. 32707096 10.1016/j.chom.2020.07.005 PMC7368656

[259] Hsu CH, Po-Liang Chen A, Chen HP, Chan YJ. Outcomes of corticosteroid treatment in critical ill adult patients with respiratory viruses-related community acquired pneumonia - a propensity-matched case control study. J Microbiol Immunol Infect. 2023;56(4):757–65. 36990896 10.1016/j.jmii.2023.02.009

[260] Sterne JAC, Murthy S, Diaz JV, Slutsky AS, Villar J, Angus DC, et al. Association between administration of systemic corticosteroids and mortality among critically ill patients with COVID-19: a meta-analysis. JAMA. 2020;324(13):1330–41. 32876694 10.1001/jama.2020.17023 PMC7489434

[261] Horby P, Lim WS, Emberson JR, Mafham M, Bell JL, Linsell L, et al. Dexamethasone in hospitalized patients with COVID-19. N Engl J Med. 2021;384(8):693–704. 32678530 10.1056/NEJMoa2021436 PMC7383595

[262] Langarizadeh MA, Ranjbar Tavakoli M, Abiri A, Ghasempour A, Rezaei M, Ameri A. A review on function and side effects of systemic corticosteroids used in high-grade COVID-19 to prevent cytokine storms. EXCLI J. 2021;20:339–65. 33746666 10.17179/excli2020-3196 PMC7975631

[263] Shang L, Zhao J, Hu Y, Du R, Cao B. On the use of corticosteroids for 2019-nCOV pneumonia. Lancet. 2020;395(10225):683–4. 32122468 10.1016/S0140-6736(20)30361-5 PMC7159292

[264] Chen F, Hao L, Zhu S, Yang X, Shi W, Zheng K, et al. Potential adverse effects of dexamethasone therapy on COVID-19 patients: review and recommendations. Infect Dis Ther. 2021;10(4):1907–31. 34296386 10.1007/s40121-021-00500-z PMC8298044

[265] Halstead ES, Umstead TM, Davies ML, Kawasawa YI, Silveyra P, Howyrlak J, et al. GM-CSF overexpression after influenza A virus infection prevents mortality and moderates M1-like airway monocyte/macrophage polarization. Respir Res. 2018;19(1):3. 29304863 10.1186/s12931-017-0708-5 PMC5756339

[266] Bekele Y, Sui Y, Berzofsky JA. IL-7 in SARS-CoV-2 infection and as a potential vaccine adjuvant. Front Immunol. 2021;12:737406. 34603318 10.3389/fimmu.2021.737406 PMC8484798

[267] Rutigliano JA, Sharma S, Morris MY, Oguin TH 3rd, McClaren JL, Doherty PC, et al. Highly pathological influenza A virus infection is associated with augmented expression of PD-1 by functionally compromised virus-specific CD8^+^ T cells. J Virol. 2014;88(3):1636–51. 24257598 10.1128/JVI.02851-13 PMC3911576

[268] Heslop HE, Leen AM. T-cell therapy for viral infections. Hematology Am Soc Hematol Educ Program. 2013;2013:342–7. 24319202 10.1182/asheducation-2013.1.342

[269] Fernández-Francos S, Eiro N, González-Galiano N, Vizoso FJ. Mesenchymal stem cell-based therapy as an alternative to the treatment of acute respiratory distress syndrome: current evidence and future perspectives. Int J Mol Sci. 2021;22(15):7850. 34360616 10.3390/ijms22157850 PMC8346146

[270] Zanirati G, Provenzi L, Libermann LL, Bizotto SC, Ghilardi IM, Marinowic DR, et al. Stem cell-based therapy for COVID-19 and ARDS: a systematic review. NPJ Regen Med. 2021;6(1):73. 34750382 10.1038/s41536-021-00181-9 PMC8575895

[271] Marano G, Vaglio S, Pupella S, Facco G, Catalano L, Liumbruno GM, et al. Convalescent plasma: new evidence for an old therapeutic tool?. Blood Transfus. 2016;14(2):152–7. 26674811 10.2450/2015.0131-15 PMC4781783

[272] Gutfraind A, Meyers LA. Evaluating large-scale blood transfusion therapy for the current Ebola epidemic in Liberia. J Infect Dis. 2015;211(8):1262–7. 25635118 10.1093/infdis/jiv042 PMC4447839

[273] Mair-Jenkins J, Saavedra-Campos M, Baillie JK, Cleary P, Khaw FM, Lim WS, et al. The effectiveness of convalescent plasma and hyperimmune immunoglobulin for the treatment of severe acute respiratory infections of viral etiology: a systematic review and exploratory meta-analysis. J Infect Dis. 2015;211(1):80–90. 25030060 10.1093/infdis/jiu396 PMC4264590

[274] Sahr F, Ansumana R, Massaquoi TA, Idriss BR, Sesay FR, Lamin JM, et al. Evaluation of convalescent whole blood for treating ebola virus disease in freetown. Sierra Leone J Infect. 2017;74(3):302–9. 27867062 10.1016/j.jinf.2016.11.009 PMC7112610

[275] Wu XX, Gao HN, Wu HB, Peng XM, Ou HL, Li LJ. Successful treatment of avian-origin influenza A (H7N9) infection using convalescent plasma. Int J Infect Dis. 2015;41:3–5. 26482389 10.1016/j.ijid.2015.10.009

[276] Luke TC, Kilbane EM, Jackson JL, Hoffman SL. Meta-analysis: convalescent blood products for spanish influenza pneumonia: a future H5N1 treatment?. Ann Intern Med. 2006;145(8):599–609. 16940336 10.7326/0003-4819-145-8-200610170-00139

[277] Joyner MJ, Bruno KA, Klassen SA, Kunze KL, Johnson PW, Lesser ER, et al. Safety update: COVID-19 convalescent plasma in 20,000 hospitalized patients. Mayo Clin Proc. 2020;95(9):1888–97. 32861333 10.1016/j.mayocp.2020.06.028 PMC7368917

[278] Agarwal A, Mukherjee A, Kumar G, Chatterjee P, Bhatnagar T, Malhotra P. Convalescent plasma in the management of moderate COVID-19 in adults in India: open label phase II multicentre randomised controlled trial (placid trial). BMJ. 2020;371:m3939. 33093056 10.1136/bmj.m3939 PMC7578662

[279] Simonovich VA, Burgos Pratx LD, Scibona P, Beruto MV, Vallone MG, Vázquez C, et al. A randomized trial of convalescent plasma in COVID-19 severe pneumonia. N Engl J Med. 2021;384(7):619–29. 33232588 10.1056/NEJMoa2031304 PMC7722692

[280] Weng J, Chen M, Fang D, Liu D, Guo R, Yang S. Therapeutic plasma exchange protects patients with sepsis-associated disseminated intravascular coagulation by improving endothelial function. Clin Appl Thromb Hemost. 2021;27:10760296211053312. 34775801 10.1177/10760296211053313 PMC8597066

[281] Patel P, Nandwani V, Vanchiere J, Conrad SA, Scott LK. Use of therapeutic plasma exchange as a rescue therapy in 2009 pH1N1 influenza A–an associated respiratory failure and hemodynamic shock. Pediatr Crit Care Med. 2011;12(2):e87–9. 20453703 10.1097/PCC.0b013e3181e2a569 PMC6328374

[282] Koch B, Schult-Dietrich P, Büttner S, Dilmaghani B, Lohmann D, Baer PC, et al. Lectin affinity plasmapheresis for Middle East respiratory syndrome-coronavirus and Marburg virus glycoprotein elimination. Blood Purif. 2018;46(2):126–33. 29698959 10.1159/000487224 PMC6008873

[283] Qin J, Wang G, Han D. Benefits of plasma exchange on mortality in patients with COVID-19: a systematic review and meta-analysis. Int J Infect Dis. 2022;122:332–6. 35709964 10.1016/j.ijid.2022.06.014 PMC9192121

[284] Martín-Loeches I, Sanchez-Corral A, Diaz E, Granada RM, Zaragoza R, Villavicencio C, et al. Community-acquired respiratory coinfection in critically ill patients with pandemic 2009 influenza A (H1N1) virus. Chest. 2011;139(3):555–62. 20930007 10.1378/chest.10-1396

[285] Morens DM, Taubenberger JK, Fauci AS. Predominant role of bacterial pneumonia as a cause of death in pandemic influenza: implications for pandemic influenza preparedness. J Infect Dis. 2008;198(7):962–70. 18710327 10.1086/591708 PMC2599911

[286] Russell CD, Fairfield CJ, Drake TM, Turtle L, Seaton RA, Wootton DG, et al. Co-infections, secondary infections, and antimicrobial use in patients hospitalised with COVID-19 during the first pandemic wave from the ISARIC WHO CCP-UK study: a multicentre, prospective cohort study. Lancet Microbe. 2021;2(8):e354–65. 34100002 10.1016/S2666-5247(21)00090-2 PMC8172149

[287] Song L, Zhao Y, Wang G, Huang D, Sai L. Analysis of risk factors associated with fatal outcome among severe fever with thrombocytopenia syndrome patients from 2015 to 2019 in Shandong, China. Eur J Clin Microbiol Infect Dis. 2022;41(12):1415–20. 36219345 10.1007/s10096-022-04506-4

[288] Wu HY, Chang PH, Chen KY, Lin IF, Hsih WH, Tsai WL, et al. Coronavirus disease 2019 (COVID-19) associated bacterial coinfection: incidence, diagnosis and treatment. J Microbiol Immunol Infect. 2022;55(6 Pt 1):985–92. 36243668 10.1016/j.jmii.2022.09.006 PMC9536868

[289] Allel K, Peters A, Conejeros J, Martínez JRW, Spencer-Sandino M, Riquelme-Neira R, et al. Antibiotic consumption during the coronavirus disease 2019 pandemic and emergence of carbapenemase-producing klebsiella pneumoniae lineages among inpatients in a Chilean hospital: a time-series study and phylogenomic analysis. Clin Infect Dis. 2023;77(Suppl 1):S20-28. 37406053 10.1093/cid/ciad151 PMC10321701

